# Advanced nanocarrier- and microneedle-based transdermal drug delivery strategies for skin diseases treatment

**DOI:** 10.7150/thno.69999

**Published:** 2022-04-11

**Authors:** Fei Qu, Rui Geng, Yijing Liu, Jintao Zhu

**Affiliations:** Hubei Key Laboratory of Bioinorganic Chemistry and Materia Medica; Hubei Engineering Research Center for Biomaterials and Medical Protective Materials; School of Chemistry and Chemical Engineering, Huazhong University of Science and Technology, Wuhan 430074, China.

**Keywords:** Microneedles, Nanocarriers, Skin diseases, Stratum corneum, Transdermal drug delivery

## Abstract

Skin diseases are the fourth leading cause of nonfatal and chronic skin diseases, acting as a global burden and affecting the world economy. Skin diseases severely impact the patients' quality of life and have influenced their physical and mental state. Treatment of these skin disorders with conventional methods shows a lack of therapeutic efficacy, long treatment duration, recurrence of the condition, and systemic side effects due to improper drug delivery. However, these pitfalls can be overcome with the applications of advanced nanocarrier- and microneedle (MN)-based transdermal drug delivery strategies that provide efficient site-specific drug delivery at the target site. These advanced transdermal drug delivery strategies can be more effective than other drug administration routes by avoiding first-pass metabolism, enhancing the drug concentration in local skin lesions, and reducing systemic toxicity. Compared with traditional transdermal delivery methods, nanocarrier- or MN-based drug delivery systems are painless, noninvasive, or minimum-invasive and require no expensive equipment. More importantly, they can introduce more advanced functions, including increased skin penetration efficiency, controlled drug release rates, enhanced targeting abilities, and theranostic functions. Here, the emergence of versatile advanced transdermal drug delivery systems for the transdermal delivery of various drugs is reviewed, focusing on the design principles, advantages, and considerations of nanocarrier- and MN-based transdermal drug delivery strategies and their applications in treating diverse skin diseases, including psoriasis, dermatitis, melanoma, and other skin diseases. Moreover, the prospects and challenges of advanced transdermal delivery strategies for treating dermatological disorders are summarized.

## Introduction

Human skin is the largest organ, acquiring an area of 20 square feet on our body surface with vital functions 1. It acts as a barrier between the outside and inside environment that prevents dangerous substances from entering the body, protects the body from infection and external elements, regulates body temperature, reduces the loss of water, and allows sensations such as touch, heat, and cold 2. Skin disease is a global public health problem that often has physiological, psychological, and social impacts. Some of the most common skin diseases include acne, alopecia, atopic dermatitis (AD), facial pigmentation, psoriasis, skin cancer, and scars. Abnormalities of the skin frequently indicate metabolic, malignant, and glandular disorders and lead to various pathological changes 3-6, including genetic, inflammatory, endocrine, hormonal, traumatic, degenerative processes, and emotions. Many skin diseases are chronic and inflammatory disorders that cannot be cured. Moreover, while most diseases affecting the skin originate in the layers of the skin, these abnormalities are important factors in the diagnosis and treatment of various internal diseases. With the deterioration of the environment and increased stress in modern life, the incidence of skin disease has risen in recent years.

Transdermal drug delivery approaches have advantages in managing skin diseases due to their characteristics of avoiding first-pass metabolism and controlling the rate of drug input over a prolonged time 7. The distinctive physiological structure of the skin presents an excellent opportunity to deliver therapeutic agents to the skin for disease treatment due to the wealth of blood vessels and lymphatic vessels in the skin, which are well connected to the rest of the body 8. Thus, skin also serves as a reservoir, thus enabling the diffusion of the penetrated drug from skin continuously over a longer period to achieve the controlled and sustained release of drug candidates that have short biological half-lives and require a high frequency of administration. Nonetheless, the skin barrier not only defends the body from pathogens but also seriously hinders the transdermal penetration of drugs. The stratum corneum (SC) of the skin is an effective barrier that limits the penetration of most drugs, making it extremely difficult for them to cross the skin 9. Overcoming SC resistance and increasing skin permeability are critical issues for improving skin disease treatment outcomes through transdermal drug delivery strategies.

Over the last decades, many approaches have been adopted to overcome the permeability barrier of the SC 10. Various chemical and physical strategies have been engineered to enhance and control the transport of a wide range of drugs across the skin, such as using chemical enhancers 11, iontophoresis 12, electroporation 13, and sonophoresis 14. However, traditional transdermal drug delivery strategies suffer from unsatisfactory delivery efficiency, high costs for expensive equipment, and painful and invasive treatment processes. Thus, there is an urgent demand for developing advanced transdermal drug delivery strategies.

Microneedle (MN) patches and nanoformulations are two advanced transdermal drug delivery methods. Compared to traditional transdermal strategies, they deliver drugs in painless and noninvasive or minimum-invasive manners, have no requirement for expensive equipment, and are feasible for self-administration. Moreover, they can have advanced functions to improve therapeutic outcomes.

Researchers have extensively investigated nanocarrier-based drug transdermal delivery systems 15, 16. Upon cutaneous administration, nanocarriers can arrive at distinct layers of the skin structure, depending on their physical and chemical properties. Nanocarriers afford a myriad of benefits for transdermal drug delivery 7, 17, including i) promoting skin penetration by disrupting lipid layers; ii) incorporating bioactive agents and diagnostic functions; iii) enhancing the stability of the loaded drugs; iv) increasing drug bioavailability by controlling pharmacokinetic profiles; v) reducing toxic side effects, and vi) achieving precise targeting. The expression of diverse surface markers and cytokines in the microenvironment of diseased skin can be considered targets for designing nanomedicine. Significantly, current advances in understanding skin disease-specific molecular pathways and functions enable novel targets to be identified, leading to new nanocarrier-based therapeutic strategies and personalized medicine. In addition, nanocarriers that respond to endogenous or external stimuli can be considered intelligent medicines with controlled or on-demand drug release properties, improving treatment outcomes 18.

As a rapidly developed transdermal technique in recent decades, MNs are composed of an array of micron-sized needles ranging from 25 to 2,000 μm that efficiently penetrate SC barriers and deliver therapeutic agents to the dermis without causing any discomfort 19. MN-assisted drug delivery is a hybrid approach that combines the benefits of both noninvasive (topical-transdermal) and invasive (injectable) drug administration approaches 20. The most significant advantage of MN is having a high skin penetration efficiency of drugs painlessly, providing excellent patient compliance and the feasibility of self-administration. Moreover, the diverse choices of needle materials can locally or systemically deliver various medicines, ranging from small molecular weight drugs, oligonucleotides, DNA, peptides, proteins, and even inactivated viruses through the skin 21. In addition, MN-based drug delivery can achieve sustained or responsive drug release, which can satisfy the demands for different treatments 22-24. Recently, MNs have been applied in interstitial fluid (ISF) sampling and signal monitoring, which can be added to the MN-based transdermal drug delivery system 25. Furthermore, combing of MNs and nanocarriers can create a more advanced transdermal delivery system with integrated advantages. Thus, nanocarrier- and MN-based transdermal drug delivery strategies show great potential in treating skin diseases.

This review first discusses the design principles of nanocarrier- and MN-based transdermal drug delivery strategies. Then, the advantages and specific design considerations of the two transdermal drug delivery strategies are introduced. After that, we highlight the applications of advanced transdermal technologies in treating various dermatological conditions, including psoriasis, superficial tumors, AD, alopecia, facial pigmentation, and scarring. Finally, an outlook is presented on the research direction and challenges of translating various advanced transdermal strategies in treating dermatological conditions.

## Design principles of nanocarrier- and MN-based delivery systems for skin diseases

**Figure 1:**
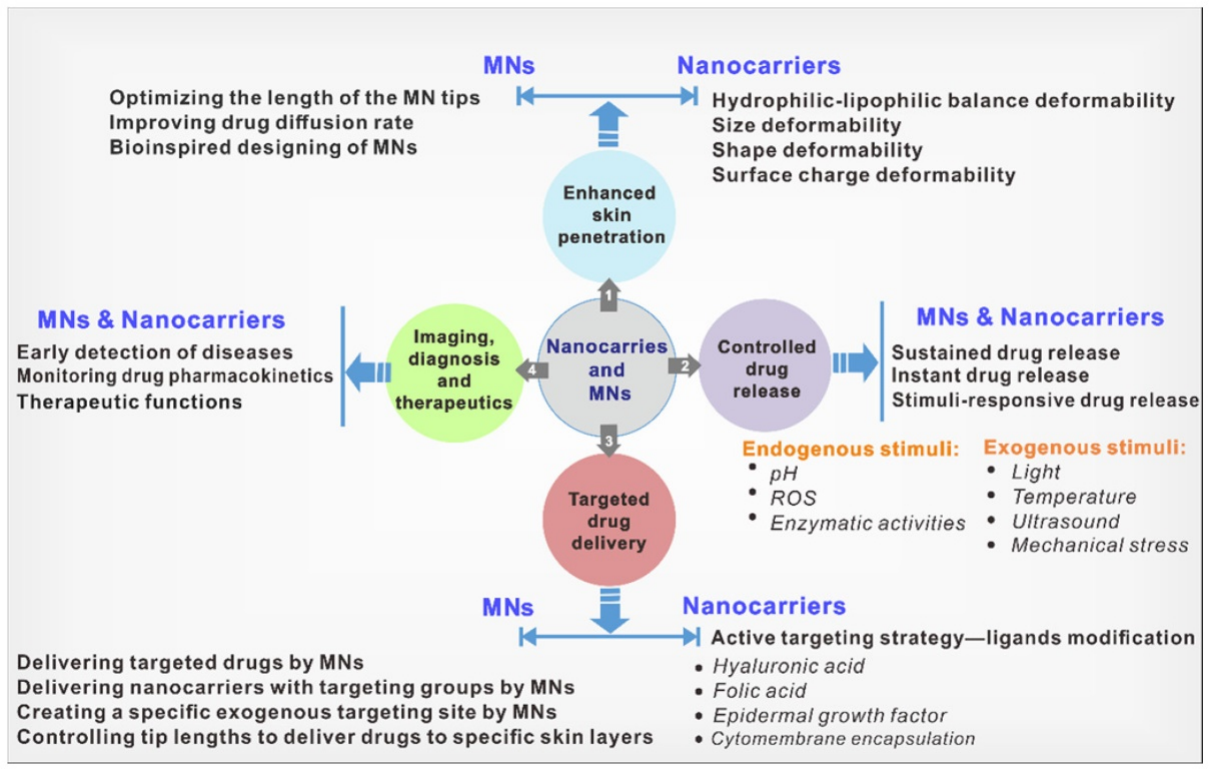
Four categories of design principles based on nanocarrier and MN delivery systems with their representative examples.

Both nanocarriers and MNs have their own merits and have been explored in numerous diseases. To realize their applications in treating skin diseases, some design principles need to be considered to improve treatment outcomes. We divided these principles into four categories: i) enhancing skin penetration; ii) controlled drug release; iii) targeted drug delivery; and iv) imaging and theranostic functions (**Figure 1**).

The **first** category of design principles is to enhance drug penetration. The outside to the inside skin consists of three primary tissues: the epidermis, the dermis, and the hypodermis 26. The barrier function of the skin strongly limits the penetration of therapeutic drugs. Even in impaired skin barrier function cases, it is challenging to deliver drugs through the outermost layer of the skin: the SC. Furthermore, certain skin diseases, such as psoriasis, have even aberrant thickening SC due to excessive proliferation of keratinocytes, one of its pathological features compared with normal skin 27. Moreover, some skin diseases, such as melanoma and basal cell carcinoma with diseased lesions below the dermis, require deeper penetration of drugs 28, 29. Therefore, there is a strong demand for enhancing the skin penetration of nanocarrier- and MN-based transdermal drug delivery strategies. It is crucial to modulate the interaction between nanocarriers and skin to better facilitate the skin penetration of nanocarriers, and studies have been carried out in tuning the physicochemical properties of nanocarriers, involving hydrophilic-lipophilic balance, size, shape, deformability, and surface charge. In the field of MNs, improved permeability can be achieved by controlling the lengths of the MN tips, optimizing the drug distribution within tips, improving the drug diffusion within skins, and using bioinspired MN designs.

The second category of design principles is controlled drug release. The primary objective of controlled drug release is to increase drug bioavailability and therapeutic outcomes. Depending on different diseases and severity, the optimal drug release rates are varied, leading to varying demands for instant drug release, sustained drug release, and stimuli-responsive drug release. Endogenous or exogenous stimuli can trigger responsive drug release. In particular, the distinct pathological characteristics of inflammatory skin or disease lesions compared with normal skin provide the chance to design skin-specific responsive drug release platforms. For example, excess reactive oxygen species (ROS) are a common hallmark of inflammatory skin diseases, such as psoriasis, melanoma, and AD 30. In skin tumors such as melanoma, there are also abnormal pH and oxidative stress of the tissue 31. Moreover, the elevated expression of certain enzymes, such as matrix metalloproteinase, has also been reported in diseased lesions 32. Exogenous stimuli include temperature, electric fields, light, and mechanical stress 33. A deep understanding of pathological characteristics and disease conditions will contribute to designing suitable nanocarrier- and MN-based transdermal drug delivery platforms.

The third category of design principles is targeted drug delivery. Enhancing drug targeting is also beneficial for improving drug bioavailability. Moreover, inflammatory skin diseases are primarily driven by inflammatory T cells, accompanied by polynuclear neutrophils, dendritic cells, and monocytes/macrophages 34. Delivering drugs that can specifically target immune cells and cytokines may achieve more precise and efficient treatment. In the field of nanocarriers, strategies to increase skin targeting are mainly active targeting strategies, which can be achieved by introducing ligands on nanocarriers for targeting specific receptors of diseased lesions (e.g., hyaluronic acid (HA), folic acid, epidermal growth factor (EGF), *etc*.) 35-37. In the field of MNs, targeting can be achieved by delivering targeted nanocarriers or targeted drugs (such as antibodies, viruses, virus-like particles, and hormones) to specific cells. In addition, the MNs themselves and the controlled tip lengths can serve as exogenous targeting sites to precisely deliver drugs.

Lastly, the fourth category is that nanocarriers and MNs can provide imaging, diagnosis, and therapeutic functions. The imaging function of nanocarriers and the diagnostic function of MNs can assist in assessing disease severity and guide the treatment. Although diagnosing these skin conditions is straightforward in everyday practice, quantifying the severity and monitoring the disease remains subjective. The imaging functions can also be used to monitor and investigate the pharmacokinetics of drugs. In addition, the compositions of nanocarriers or MNs, in addition to drugs, can have intrinsic therapeutic functions 38. All of these can contribute to improving treatment outcomes.

The following section will describe the specific design of nanocarriers and MNs for the enhanced skin penetration of drugs, controlled drug release, enhanced targeting strategies, and imaging and theranostic functions to achieve greater therapeutic benefits in skin diseases.

## Nanocarrier-based transdermal drug delivery strategies

Nanocarriers-based transdermal drug delivery systems offer multiple advantages over conventional modes, such as enhanced physicochemical stability of drugs, increased skin permeation, improved biodistribution, effective targeted accumulation, and controlled drug delivery. Especially, nanocarriers can be applied in the hair follicle (HF)-targeted delivery to enhance the topical treatment outcomes of some appendage-related skin diseases, such as alopecia and acne 39, 40. Moreover, nanocarrier-based drug delivery systems with combined imaging modalities and intrinsic therapeutic functions can improve treatment outcomes.

The most widely explored topical nanocarriers for drug delivery include liposomes, inorganic nanoparticles (NPs), solid lipid NPs (SLNs), nanoemulsions, microemulsions, nanogels, dendrimers, micelles, and others. The percutaneous absorption of nanocarriers is dominated by their structure and surface properties. They can be designed to interact with the skin to increase penetration or modified to perform different functions, including controlled drug release, effective targeting, and imaging. In the following section, we shed light on the various design features of nanocarriers that have been exploited for topical applications.

### Enhanced skin penetration

The skin penetration efficiency of nanocarriers is a key parameter for determining their transdermal treatment outcomes. The physicochemical properties of nanocarriers such as hydrophilic-lipophilic balance, size, shape, deformability, and surface charges have been shown to affect their penetration in highly complex skin by modulating the nano-skin interactions 41. Thus, exploring and understanding the nano-skin interactions is crucial for the successful clinical applications of nanocarriers in many skin diseases. In this section, we will clarify the design principles of physicochemical properties of nanocarriers for enhancing skin penetration.

#### Hydrophilic-lipophilic balance of nanocarriers

Increasing the lipophilicity of hydrophilic drugs is an important option for enhancing their transdermal delivery efficiency due to the skin lipid composition. Among various strategies, encapsulating hydrophilic drugs in nanocarriers is a more general and effective method than other traditional chemical enhancing methods, such as prodrug modification and using chemical enhancers.

**Figure 2:**
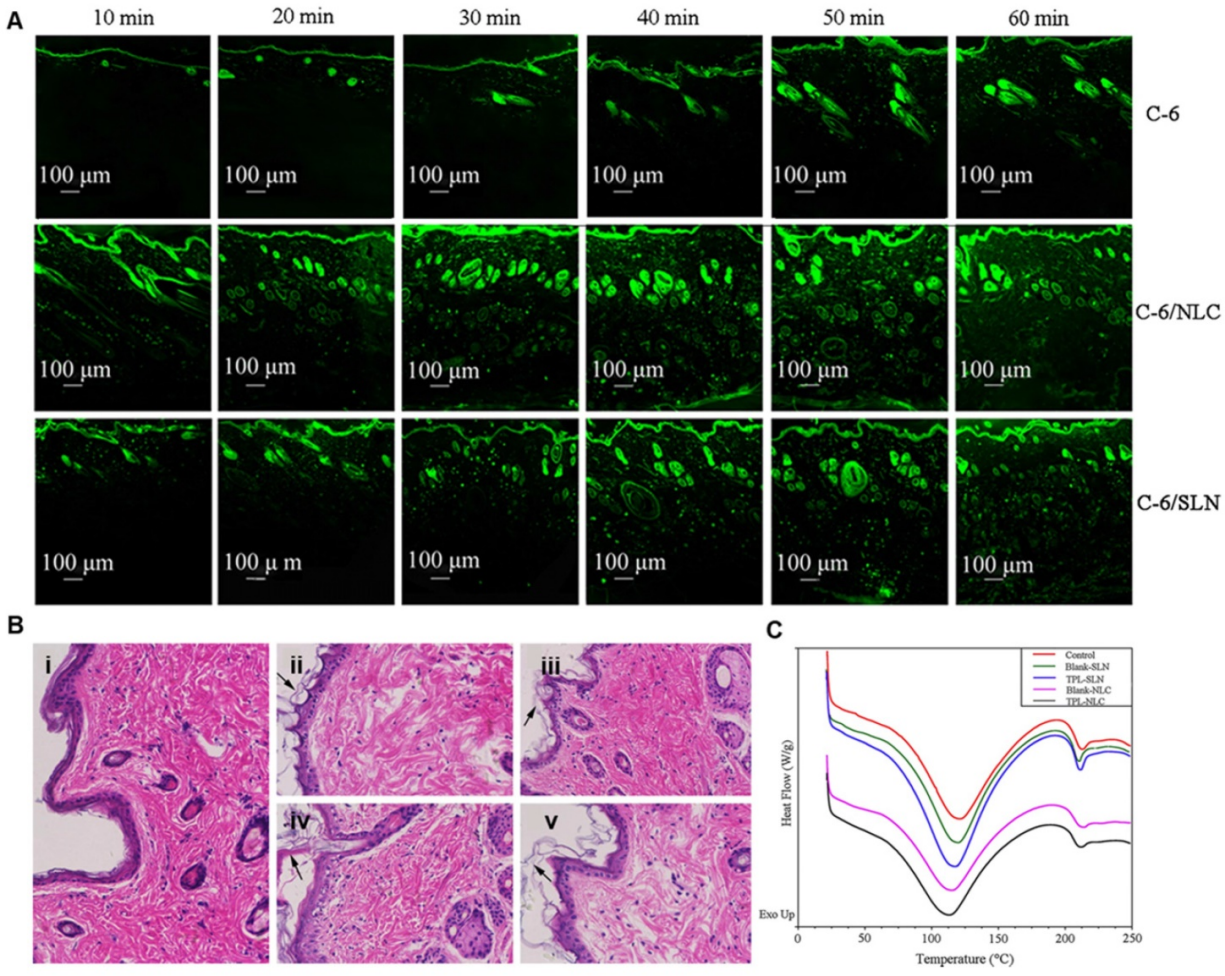
Skin distribution of NPs. (A) Confocal laser scanning microscopy images (100×) of skin samples treated with free coumarin-6, coumarin-6/nanostructured lipid carries (NLC), and coumarin-6/SLN. (B) Histopathological photomicrographs (200×) of skin treated with (i) normal saline; (ii) Blank-NLC; (iii) Blank-SLN; (iv) TPL-NLC; (v) TPL-SLN. (C) The differential scanning calorimetry (DSC) thermograms of skin tissue were treated with normal saline, Blank-NLC, Blank-SLN, TPL-NLC, and TPL-SLN. Adapted with permission from reference 46. Copyright 2018 Springer Nature.

The homology between lipid nanomaterials and epidermal lipids makes lipid nanocarriers the first choice for transdermal drug delivery. Compared with polymer nanocarriers, lipid nanocarriers (formulated by oily triglyceride and soybean lecithin) showed approximately twice the flux rate of ibuprofen in transdermal delivery 42. The enhanced transdermal delivery of lipid-based nanocarriers was ascribed to their ability to enhance skin hydration and disruption of the SC layer. Moddaresi *et al.* reported that lipid nanocarriers could enhance skin hydration without causing changes in skin viscoelasticity 43. Moreover, lipid nanocarrier hydration allows the drug to penetrate deeper skin layers by reducing the accumulation of keratinocytes 44. The amphiphilic composition also promotes the distribution of the drug in the skin. For example, Lohan *et al.* found that nanosized lipid particles increased the amount of dexamethasone in SC by two times compared with commercial cream 45. Gu and coworkers explored the interactions between skin and different triptolide-loaded lipid nanocarriers (TPL-NPs) to further understand the mechanisms of penetration enhancement and transport properties of TPL-NPs 46. As shown in **Figure 2A**, TPL-NPs penetrated skin in a time-dependent process and accumulated in the HFs and even throughout the skin after 1 h. The histopathological analysis of skin structure showed that the application of TPL-NPs led to thinner SC, thicker epidermis, and larger intercellular spaces, indicating that TPL-NPs could disrupt skin structure (**Figure 2B**). The differential scanning calorimetry (DSC) curves of the skin samples demonstrated that the melting point of keratin in the TPL-NPs group was significantly lower than that in the control group (**Figure 2C**). Therefore, the enhanced TPL penetration could be attributed to the changes in the structure and thermodynamic activity of the SC by TPL-NPs.

#### Size and deformability of Nanocarriers

**Figure 3:**
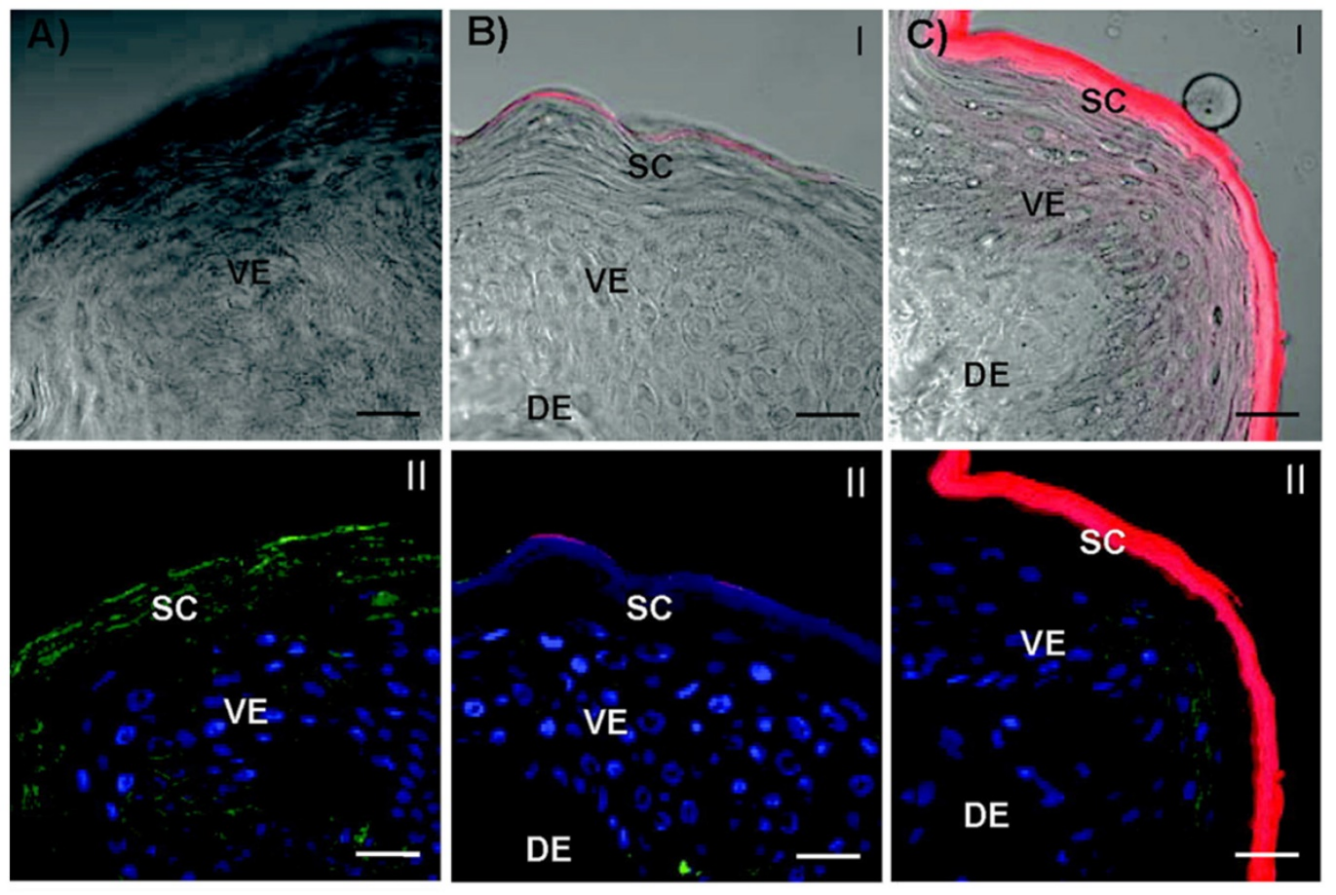
Confocal laser scanning microscopy images of the skin cross-sections of microtomed porcine skin layers after treatment with dendrimers. (A) Vehicle (ddH_2_O) control. (B) G4-RITC-NH_2_. (C) G2-RITC-NH_2_. (dendrimer conjugates (red), cell membrane stained by WGA-AF488 (green), and nuclei stained by DAPI (blue)). Scale bar: 10 μm. Adapted with permission from reference 48. Copyright 2012 American Chemical Society.

The size of nanocarriers is another important factor that influences transdermal drug delivery. Nanocarriers of different sizes enter the skin through different transdermal routes. Normally, smaller nanocarriers have deeper skin penetration due to their stronger diffusive ability. For example, Takeuchi *et al.* studied the skin permeability of poly(lactic-co-glycolic) acid (PLGA) NPs of different sizes in iontophoresis 47. The fluorescence microscopy images illustrated that 50-nm PLGA NPs accumulated more in HFs and intercellular space than 100-nm PLGA NPs. Poly(amidoamine) (PAMAM) dendrimers were reported to be effective skin penetration enhancers. Yang *et al.* carried out a series of studies of dendrimer-skin interactions 48. In terms of size, smaller PAMAM dendrimers (generation 2 (G2)) were found to have higher skin permeation than larger dendrimers (G4) (**Figure 3**). Su *et al.* showed that the different sized nanoemulsions entered skins through different mechanisms. The nanoemulsions below 80 nm could permeate the viable epidermis and fill in the whole hair follicles, whereas bigger nanoemulsions, such as 500 nm, only migrate along the hair follicles 49.

However, there are some exceptions to this size-dependent transdermal efficiency when nanocarriers are deformable. For example, some studies reported that skin permeability was inversely related to the sizes of lipid nanoemulsions when their sizes were below 50 nm 50. The fluidic nature of lipids in lipid nanoemulsions provides strong deformability and may penetrate the skin barrier in arbitrary shapes. Larger nanoemulsions could even recover quickly after elastic deformation through narrow intercellular gap junctions. Similarly, the transferosomes deformed by strong shearing can completely cross the SC through small aqueous channels between keratinocytes and even enter the dermis to reach systemic circulation 51.

#### Shape of Nanocarriers

The shape of nanocarriers also influences their skin penetration ability, but the mechanism still needs to be further explored. Many related studies have used rigid NPs to study the shape effects on transdermal delivery. In one study, the skin penetration of spherical and rod-like gold NPs (AuNPs) was visually and quantitatively evaluated using transmission electron microscopy (TEM), two-photon photoluminescence microscopy (TPPL), and inductively coupled plasma-optical emission spectrometry (ICP-OES) 52. The results indicated that the content of gold nanorods (AuNRs) in the skin was significantly higher than that of gold nanospheres in mouse skin. Thus, shape is an important factor in determining the penetration depth of NPs in skins, in addition to size. Similarly, Hsiao *et al.* reported that the deposition of AuNRs in the epidermis was 1.9 times that of gold nanospheres, and the accumulation of AuNRs in the subcutaneous adipose tissue, a marker for skin penetration, was 1.7 times that of spheres 53. Some studies have shown that Au nanostars have higher follicular penetration than spheres 54. More studies have to be done to reveal the effects of nanocarriers' shape on transdermal delivery.

#### Charge on Nanocarriers

The surface charge of nanocarriers is a key parameter in mediating NP-lipid membrane interactions, yet which type of charge promotes skin absorption remains controversial. According to the “Donnan exclusion effect”, positively charged nanocarriers should interact better with negatively charged skin cells 55; thus, many studies have shown that positively charged NPs have better skin penetration. For example, Qin *et al.* investigated the charge effect of liposomes on skin penetration in a nude mouse model by conjugating liposomes with 4-cholesterocarbonyl-4′-(N,N,N-triethylamine butyloxyl bromide) azobenzene (CAB, positive) and 4-cholesterocarbonyl-4′-(N,N-diethylamine butyloxyl) azobenzene (ACB, neutral) 56. As the fluorescent probe, CdTe quantum dots (QDs) were encapsulated in liposomes. *In vivo* skin delivery results showed that the cationic CAB liposomes showed better transdermal delivery efficiency. In 2 h, cationic CAB liposomes delivered QDs to the upper epidermis, whereas QDs in neutrally charged ACB liposomes remained on the skin surface with no apparent penetration. Ibaraki *et al.* also observed similar results that cationic liposomes have the most effective skin permeability compared with anionic or neutral liposomes 57. In addition to liposomes, it has been shown that other cationic organic nanocarriers, such as PAMAM, generation 3 to generation 6 dendrimers, and cationic AuNPs showed better skin penetration than neutral and negative nanocarriers 58-60. Conversely, some reports claimed that negatively charged nanocarriers are more beneficial to skin delivery than positively charged nanocarriers. The enhanced penetration may be attributed to the generation of temporary skin channels caused by the repulsive force between negative nanocarriers and negative skin lipids. Maione-Silva *et al.* found that liposomes with negative surface charges can promote ascorbic acid cutaneous permeation 61. Ternullo *et al.* investigated the charge effect of human epidermal growth factor (hEGF)-loaded deformable liposomes (DLs) on skin penetration 62. Anionic DLs exhibited the highest hEGF retention in human skin compared to neutral and cationic DLs. However, Tupal *et al.* studied the skin delivery of different doxorubicin (DOX)-loaded SLNs 63. The results indicated that neutral SLNs are more effective at transporting DOX into the skin than both positive and negative SLNs. They believed that the “Donnan exclusion effect” could only improve the partial penetration of positively charged SLNs into deeper skin layers, while the penetration of negatively charged SLNs is blocked due to its repulsion with the SC. Subongkot *et al.* reported similar results 64. They observed that neutrally charged microemulsions significantly enhanced the skin penetration of celecoxib.

### Controlled drug release

Traditional drug transdermal delivery often leads to systemic side effects due to uncontrolled drug burst release. Advanced nano drug delivery systems can be designed to deliver low-bioavailability agents in a controlled manner to achieve appropriate therapeutic doses at target sites. The controlled release systems of transdermal nanocarriers reported so far are mainly divided into two release modes: sustained release and stimuli-responsive release.

Sustained-release preparations are often preferred in clinical medication, which provide decreased dosing frequency, stable blood concentration, and a prolonged medication cycle 65. Compared with small molecular drugs, nanocarriers can increase the drug retention time in skins due to their slower clearance rate. Moreover, the nanocarriers can be specially designed to have stronger interactions with skins for prolonged drug release. Castleberry *et al.* developed a polymer-drug conjugate system (PATRA) for the topical delivery of all-trans retinoic acid (ATRA) by coupling hydrophobic ATRA to hydrophilic polyvinyl alcohol (PVA) *via* an ester bond 66. The amphiphilic PATRA formed micelles that protected ATRA from UV degradation. PVA has been reported to interact with mucosal proteins *via* hydrogen bonding. The resulting adhesive properties can prolong the residence of drugs in the skin. As a result, drug release studies showed that PATRA sustainably released active ATRA for up to 10 days *in vitro*, and dermal retention of the coupled ATRA was significantly increased over that of free ATRA in explant pig skin.

Drug release from polymeric nanocarriers can also be controlled by modulating the surface wettability of polymers. Achieving tunable surface wettability by providing unique micro/nanoscale topologies through electrospinning technology has attracted attention. For example, Adepu *et al.* investigated the effects of the surface wettability of micropatterned drug-loaded electrospun cellulose acetate nanofibers on the transdermal release of diclofenac sodium 67. The variation in wettability was obtained from patterning-induced surface hydrophobicity, resulting in the controlled release of the hydrophilic drug. *In vitro* skin permeation study showed that the transdermal system with optimized micropatterned dimensions achieved a zero-order sustained drug release for up to 12 hours compared to nonpatterned samples, suggesting that this patterning-assisted method can potentially control the initial burst release of drugs with a low half-life.

In addition to long-term delivery, on-demand drug release is a common requirement in many therapeutic applications. On-demand drug release can be triggered by exogenous stimuli such as light, temperature, or endogenous stimuli such as pH, ROS, or enzymatic activities 68, 69.

There have been limited reports regarding the use of endogenous stimuli-responsive drug release in nanocarrier-mediated transdermal drug delivery. The different pH values between normal and disordered skin can be used as stimuli to design nanocarriers. The acidic mantle on the skin surface (pH ~ 5.5) is essential for maintaining an effective barrier and homeostasis 68, 70. Disruption of this pH condition causes several skin diseases, such as AD (pH 7 ~ 9). Park *et al.* designed a pH-responsive hydrogel (cl-CMC-g-pHEA) from carboxymethyl cellulose (CMC) and 2-hydroxyethyl acrylate (2-HEA) that was specifically designed for the pH change in AD skins for transdermal delivery of naringenin, a drug for the treatment of AD 68. The swelling ratios of all hydrogels at pH 7.5 and 8.5 are higher than those at pH 5.5 due to the electrostatic repulsion caused by the ionization of the carboxyl groups. The release kinetics of naringenin from the hydrogel could be analyzed by the Fickian diffusion mechanism. The cumulative drug release at pH 5.5, 7.5, and 8.5 after 24 h was 42%, 70%, and 73%, respectively. *In vitro* transdermal experiments showed that the cl-CMC-g-pHEA hydrogel enhanced the skin penetration of naringenin. Taken together, this pH-responsive cl-CMC-g-pHEA hydrogel presents great application possibilities as a transdermal delivery carrier for the treatment of various skin diseases generated by pH imbalance.

Nanocarriers can also be designed to release drugs in response to environmental changes such as light, temperature, and ultrasound 71-73. Temperature-sensitive polymers are considered one of the most popular stimuli-responsive materials for drug release. Kim *et al.* synthesized spherical hydrogel beads by loading dexamethasone and magnetite NPs in a temperature-responsive poly(N-isopropylacrylamide-co-vinyl-2-pyrrolidinone) hydrogel (p(NIPAm-co-VP)), which changed its solubility at a lower critical solution temperature 74. Furthermore, they incorporated drug-loaded hydrogel beads into a biocompatible hydrogel to develop a transdermal patch. The magnetite NPs dispersed in the hydrogel beads absorbed visible light to generate heat, resulting in shrinkage of the temperature-responsive hydrogel proportional to light intensity and on-demand drug release.

Most reported temperature-sensitive systems have a responsive temperature of approximately 40 °C. This localized overheating may cause damage to healthy tissues. Because cooling is safer to handle than heating, the cooling-triggered release may be more beneficial for certain skin diseases. Vikulina *et al.* prepared poly(N-isopropylacrylamide) (pNIPAM) microgels (MM-gels) by hard templating and cross-linking of the hydrogel using mesoporous crystals of vaterite CaCO_3_ 75. When heated above the lower critical solution temperature, the MM-gels shrunk by a factor of approximately two at neutral pH and to a much higher extent under acidic skin conditions. The study demonstrated that macromolecular dextrans could be spontaneously captured into the microgel by pNIPAM when the temperature increased from 22 to 35 °C, followed by dextran release upon further cooling the microgel to 22 °C. This heating-induced encapsulation and cooling-induced release transdermal delivery system may pave the way for some skin disease applications in the future.

Ultrasound has been demonstrated as a potential skin penetration enhancer, and its mechanism of mediating drug therapy has been well explored 76. Anirudhan* et al.* reported a temperature and ultrasound dual-responsive transdermal drug delivery system based on mesoporous silica NPs (MSNs) with grafted copolymer chains of amino ethyl methacrylate (AEMA) and 2-tetrahydropyranyl methacrylate (2-THPMA) 77. 2-THPMA has an ultrasound cleavable acetal linkage that can change from a hydrophobic molecule into a hydrophilic molecule upon exposure to ultrasound. These copolymers on MSNs exhibited an open conformation below 4 °C, allowing for maximum drug loading into MSNs through the pores, and collapsed at physiological temperature to close the pores. The copolymers on MSNs decomposed upon ultrasound exposure, thereby allowing controlled drug release and skin permeation.

### Targeted Drug Delivery

Since the long-term application of many chemotherapeutic drugs has severe local and even systemic side effects, development of more precise treatment strategies can be very important for reducing drug doses. Designing tissue- or cell-targeting drug delivery systems is critical for achieving precise treatment. Nanocarrier systems display great potential in targeted drug delivery due to their flexible surface modifications. Compared with intravenous administration, the transdermal delivery route bypasses the vascular system with complex protein and cell components and blood clearance by the mononuclear phagocyte system, which makes targeting design more efficient.

The targeted skin delivery of nanocarriers explored so far is mainly an active targeting strategy. Introducing targeting ligands aims to enhance the transdermal delivery efficiency or increase the skin retention of drugs.

**Figure 4:**
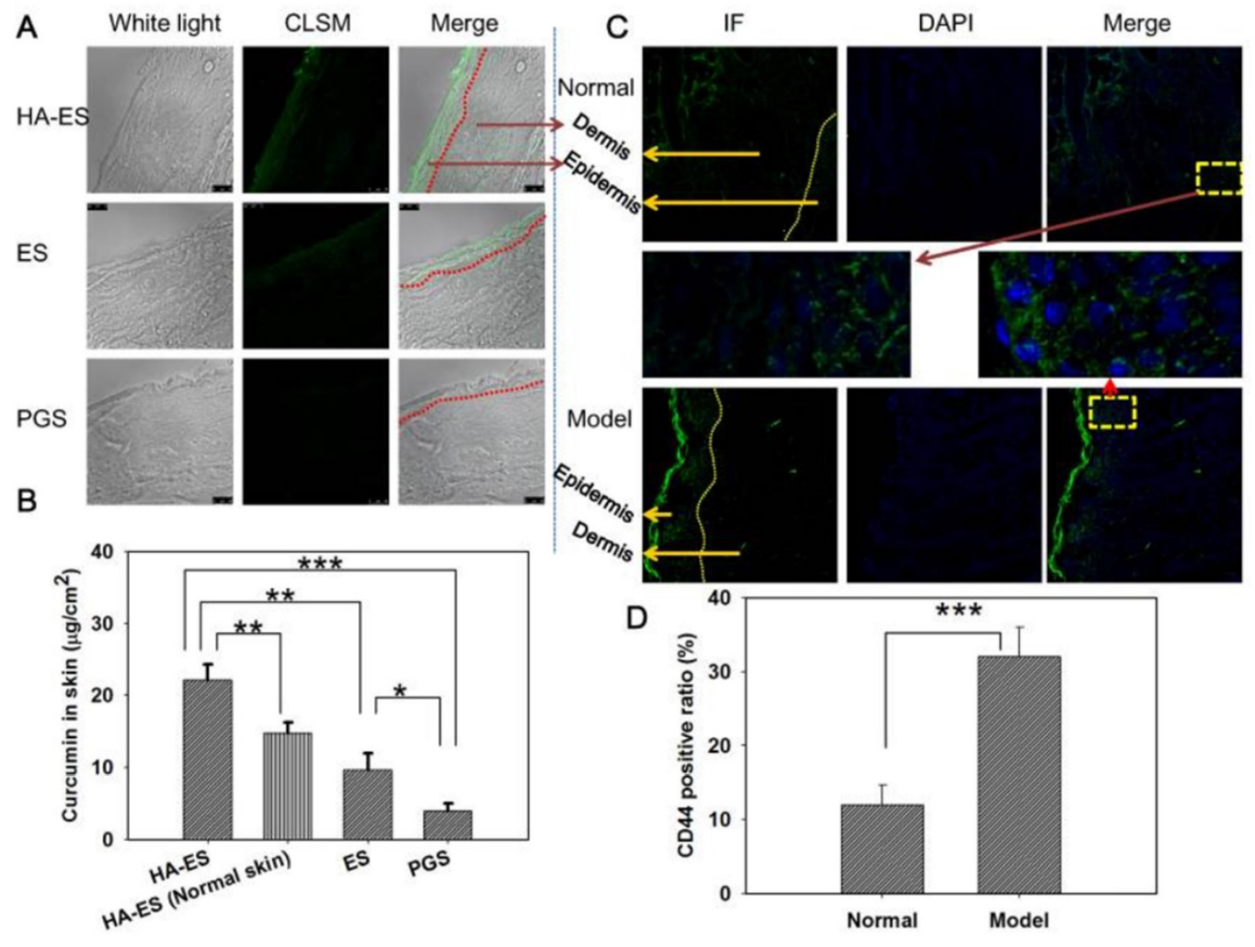
(A) Confocal laser scanning microscopy image (200×) of curcumin fluorescence in frozen psoriasis-like mouse skin sections and (B) drug skin retention detected by HPLC (n=5) after treatment with the various curcumin formulations for 8 h. (C) The immunofluorescence images (200×) and (D) semi-quantitative analysis (n=5) show the distribution of CD44 expression in psoriasis-like and normal mouse skin. *p < 0.05, **p < 0.01, ***p < 0.001. Normal, mice treated without any formulations; Model, treated with IMQ only; HA, hyaluronic acid; HA-ES, curcumin-loaded HA-modified ethosomes; ES, curcumin-loaded ethosomes; PGS, curcumin 25% propylene glycol solution; IMQ, imiquimod ointment; IF, immunofluorescence. Adapted with permission from reference 79. Copyright 2019 Ivyspring International Publisher.

The targeted skin delivery of nanocarriers explored so far is mainly an active targeting strategy. Introducing targeting ligands aims to enhance the transdermal delivery efficiency or increase the skin retention of drugs 78. Studies have found that the CD44 protein is highly expressed in certain skin diseases such as psoriasis. HA is a natural ligand of the CD44 protein and can potentially be used to construct targeted nanodrug transdermal delivery systems. Zhang *et al.* developed curcumin-loaded HA-modified ethosomes (HA-ES) for topical psoriasis treatment 79. The HA-ES system's specific targeting of the CD44 protein increased curcumin accumulation in inflamed skin (**Figure 4**).

**Figure 5:**
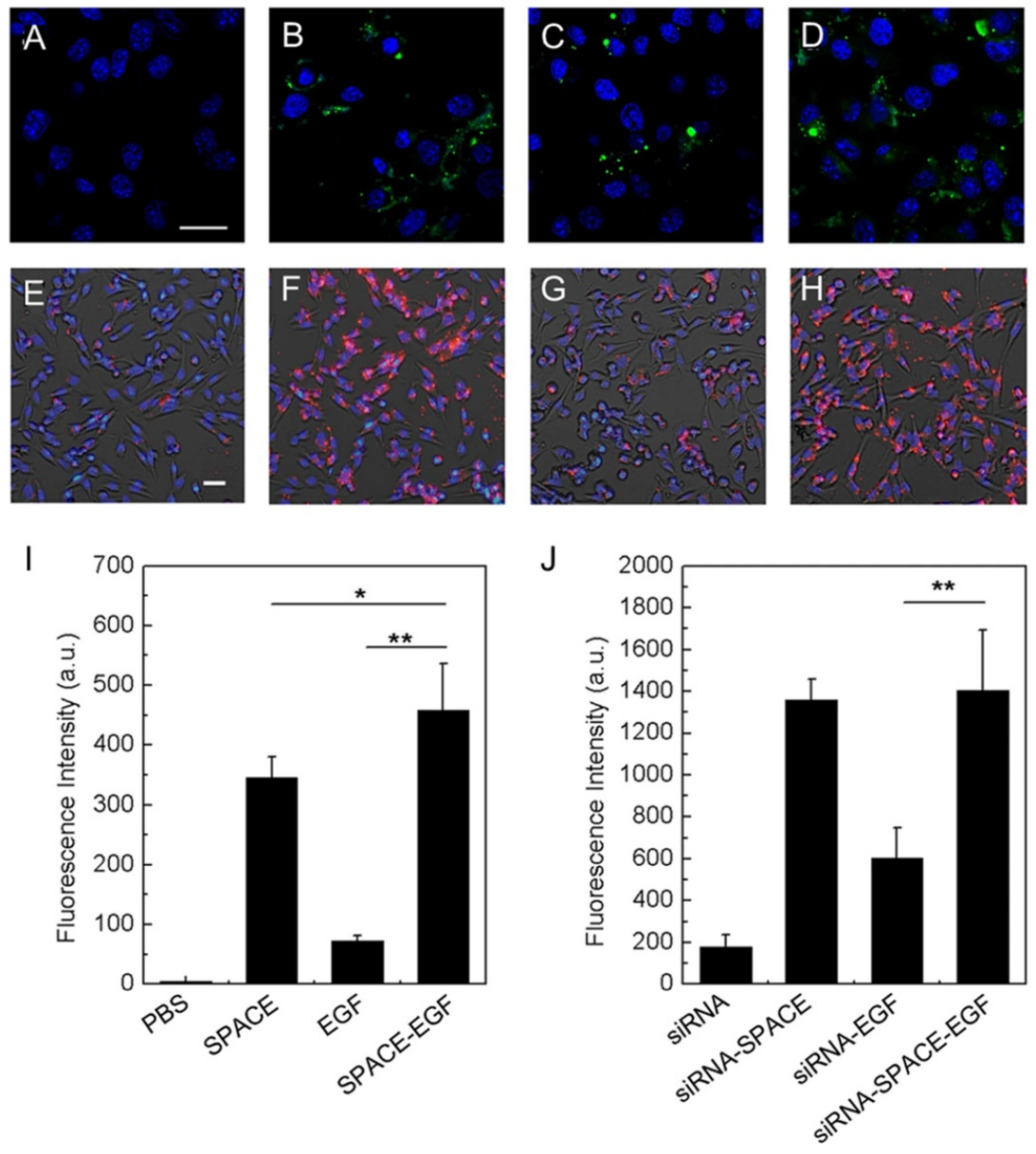
Cell penetration of SPACE-EGF and SPACE-EGF-siRNA. (A-D) Cells were treated for 6 h with PBS, SPACE, EGF, and SPACE-EGF, respectively. (E-F) Cells were incubated for 6 h with siRNA, siRNA-SPACE, siRNA-EGF, and siRNA-SPACE-EGF, respectively. (I, J) The mean fluorescence intensities in melanoma cells after 6 h were compared. SPACE was also labeled fluorescently. Scale bar: 20 μm. Adapted with permission from reference 80. Copyright 2016 Springer Nature.

EGF can specifically bind to the EGF receptor overexpressed in many cancer cells such as melanoma cells, which can be used in targeted drug delivery. Ruan *et al.* developed a fusion peptide nanocarrier that was a skin-penetrating peptide and cell-targeting peptide SPACE modified with EGF for the topical delivery of siRNA to melanoma cells 80. *In vitro* experiments indicated that the EGF motif significantly improved the melanoma cell targeting ability of the SPACE-EGF-siRNA complex (**Figure 5**).

In recent years, some other ligands have also been considered as potential targeting strategies. For example, Huang *et al.* designed BQ-788 (an antagonist selectively binding to endothelin ET_B_ receptor on the cell membrane of melanocytes) grafted ZnO QDs for transdermal delivery of a tyrosinase inhibitor 81. The BQ-788/ZnO QDs could specifically target melanocytes and were taken up within 1 h. Gu *et al.* synthesized an *Escherichia coli* (*E. coli*)-derived outer membrane vesicle (TEV) modified with a peptide sequence RWrNMGGGGIVRRADRAAVP for transdermal and tumor-targeting delivery 82. This peptide exhibited a high affinity for integrin αvβ3 overexpressed in cancer cells. TEVs were shown to have a specific accumulation in melanoma cells.

### Imaging, diagnosis, and therapeutics

Topical application of drug-loaded nanocarriers is an attractive strategy for treating skin diseases. Simultaneously, nanocarriers with other functional modules can be created for improving treatment outcomes. For example, nanoplatforms with imaging functions enable the study of the transdermal mechanism of nanocarriers and the evaluation of the treatment effects in real-time.

Lee *et al.* developed a hyaluronate-AuNRs/death receptor 5 antibody (HA-AuNR/DR5 Ab) complex for the transdermal treatment of skin cancer 83. DR5 Ab can specifically target death receptor 5 (DR5), which is overexpressed on the cancer cell membrane, to induce apoptosis of cancer cells. The effective transdermal delivery of the HA-AuNR/DR5 Ab complex could be confirmed by the photoacoustic imaging functions of AuNRs. Zhang *et al.* designed a multifunctional transdermal nanoplatform (+)T-SiDs for magnetic resonance imaging (MRI)/Near-infrared (NIR) dual-modal imaging-guided chemo-photothermal therapy (PTT) of superficial tumors 84. The (+)T-SiDs utilized superparamagnetic iron oxide (SPIO) NPs as the cores to load DOX, and the surfaces were modified with cationic phospholipids, the transdermal enhanced peptide TD (transdermal peptide, ACSSSPSKHCG), and the NIR dye 1,1'-dioctadecyl-3,3,3',3'-tetramethylindotricarbocyanine iodide (DIR). The TD and cationic lipids enhanced the transdermal delivery of (+)T-SiDs. The MRI and NIR imaging results showed that (+)T-SiDs exhibited efficient transdermal permeation and tumor accumulation.

The study and evaluation of the skin-nano interactions require even more advanced designs of nanocarriers, such as having responsive imaging signals to observe the drug release from nanocarriers. Shi *et al.* developed curcumin-loaded nanocarriers that contained BODIPY-based dyes to study their skin-entry mechanism. The dyes emitted NIR fluorescence when they were encapsulated in nanocarriers, but “turned off” the emission when they were released from the nanocarriers and aggregated in water due to the “aggregation-caused quenching” (ACQ) effect. By using this property, it was demonstrated that the intact nanocarriers could not enter the skin. Instead, they could filtrate into the SC and accumulate in hair follicles to release drugs and improve skin permeability 85.

In addition, it has been demonstrated that some nanomaterials have intrinsic therapeutic effects on skin diseases 86. For example, Han *et al.* presented the self-therapeutic effects of topical-applied alkyl-terminated AuNPs on psoriasis 86. Thus, we can hypothesize that combined treatment effects from encapsulated drugs and certain nanocarriers can potentially lead to more effective therapeutics. Up to now, the applications of multifunctional nanoplatforms in dermatology are still at the early stage and need to be further developed.

## MN-based drug delivery strategies

In 1998, Henry *et al*. 87 first used silicon MNs to enhance drug penetration in the skin. Compared with traditional penetration methods such as ultrasound-mediated penetration and iontophoresis, which produce nanoscale cracks in the skin SC, MNs can pierce the SC to produce visible microscale pores in the skin that enhance the transdermal efficiency of small molecular drugs and even macromolecules 88. Moreover, the application of MNs does not cause skin damage, as the pore channels generated by MNs quickly close due to the elastic retraction force of the skin. MNs with different geometries and sizes can be made from a variety of materials, including silicon, metals, and polymers 89. Especially, polymer MNs can release encapsulated drugs from dissolvable or degradable needle tips, which reduces the risks of leaving metal tips in the skin. Thus, the following sections focus on polymer MNs. Moreover, the encapsulation of nanocarriers in MNs can make a more advanced transdermal drug delivery platform by combining the advantages of both parts. On the one hand, MN-based drug delivery systems have intrinsically higher transdermal efficiency than nanomaterials. The design of nanocarriers delivered by MNs can be more flexible without worrying about their transdermal properties, providing ways to enhance targeting, responsive, and theranostic properties. On the other hand, the rich and tunable chemical and physical properties of both MN tips and encapsulated nanomaterials provide a greater ability to control pharmacokinetics and offer more treatment options.

In this part of the review, we introduced the considerations of designing MN-based drug delivery strategies, including delivery nanocarriers, to achieve better treatment outcomes for skin disease. These considerations included improving skin penetration efficiency, enabling sustainable drug release and precise targeting, and designing a theranostic transdermal drug delivery platform.

### Enhanced skin penetration and drug diffusion

One of the most significant advantages of MNs is the direct delivery of drugs into diseased skin to enhance treatment efficiency. Ideally, all drugs in the needles should be forced into the skin. However, the actual amount of penetrated drugs being penetrated was usually less than 100% because of needle fracture or incomplete insertion. Moreover, drugs injected by MNs usually depend on passive diffusion to distribute in the skin. However, the diffusion of drugs in skins is limited due to the crowded skin environment, which negatively impacts treatment outcomes. Therefore, enhancing the skin penetration and diffusion of drugs are essential issues to be considered.

In the recent decade, significant progress has been made to facilitate the insertion capability and skin penetration of MNs. The skin penetration of MNs depends on many different factors, such as application force and skin elasticity. Because skins are elastic, some MNs push the skin instead of penetrating and creating holes to deliver the drug cargo. To solve this problem, Lee *et al*. fabricated MNs with micropillars to distribute the application force uniformly over each MN tip, thus facilitating a complete insertion of needle tips into the skin 90. In addition, pedestal-based MNs with an extended needle length can counteract the skin's elasticity and have additional mechanical strength for deeper penetration/insertion. Chen* et al*. designed a similar MN system by introducing a dissolvable support array between MN bases and needles, providing extra length for complete insertion of the MNs 91.

To enhance the permeability of drugs through the epidermis, bubble-generating MNs have been utilized for the transdermal delivery of drugs. The formation of such microbubbles evokes a unique vortex flow field leading to a powerful, autonomous "pump-like" action and locally applied forces, resulting in active and exceedingly efficient transport and penetration of the embedded therapeutic payload. For example, Lopez-Ramirez* et al*. designed an autonomous, biodegradable, dynamic MN delivery platform that employed magnesium microparticles loaded within an MN as built-in engines for deeper and faster intradermal payload delivery 92. The magnesium microparticles react with ISF, resulting in the rapid production of explosive-like H_2_ bubbles, supplying the necessary force to breach the skin barrier and enhance payload delivery.

Bioinspired designs, such as those that mimic animal organs, can also be employed to improve the performance of MNs for successful skin insertion. In one study, Masato *et al*. designed an MN aimed at painless blood collection by biomimicking a mosquito 93. They fabricated a three-piece MN mimicking a mosquito needle (a blood-sucking central hollow labrum and two side solid maxillae having jagged edges) by an ultra-precision 3D laser lithography system. The developed MN was divided into two parts that can move independently and successfully inserted into an artificial skin. When the two halves are alternately advanced during insertion, the puncturing force applied to the skin is reduced. It was experimentally confirmed that this needle successfully penetrates the skin, and such a design could decrease the force required to insert (and remove) the MNs.

### Controlled drug release

The demanded drug release rates from MNs may vary depending on different categories and conditions of skin diseases. Achieving controlled drug release from MNs could optimize therapeutic outcomes, especially when multiple drugs were simultaneously loaded into MNs. Controlled drug release can be divided into sustained drug release, instant drug release, and responsive drug release, which can be achieved by modifying the degradation and swelling profile of the host polymer or nanocarriers, tuning the diffusion profile of the encapsulated drug, and using responsive materials.

Instant or burst drug release is necessary and desirable in some diseases requiring immediate action of drugs, such as analgesia and tissue repair. In these cases, dissolving MNs (DMNs) are used to encapsulate drugs that release drugs when the needles are dissolved by skin ISF. For example, our group fabricated methotrexate (MTX)-loaded MN patches made of HA, which completely dissolved in 10 minutes after being inserted into the skin 94.

Many chronic diseases require the sustained release of drugs. The aim of sustained drug delivery using MNs is to maintain a steady-state drug concentration for a specific period and reduce side effects. In MNs, polymer swelling and degradation are the primary mechanisms for achieving sustained drug release 95. Especially, hydrophobic and biodegradable polymers, such as PLGA, are preferred for making MNs with sustainable drug release. For this kind of MN, a quick separation between MN tips and bases is necessary. Li* et al*. fabricated MNs with PLGA through a micromolding method to slowly release levonorgestrel for ~1 month *in vitro* 96.

As a unique design, sodium bicarbonate and citric acid were loaded into the MN patch backing, which reacted in contact with ISF to form carbon dioxide bubbles to separate of MN tips from the bases within 1 min of skin insertion.

The combination of sustained-release and instant release from MNs can also be necessary, especially when multiple types of drugs are loaded. For instance, Yang *et al*. developed a bilayer MN to rapidly release triamcinolone acetonide (TAA) and sustainably released 5-fluorouracil (5-Fu) to achieve efficient hypertrophic scar treatment 97. Specifically, the needle tip layer made from chitosan and dextran slowly released 5-Fu. The tail layer, which contained HA as an excipient and hydroxypropyl-β-cyclodextrin (HP-β-CD) as the inclusion of insoluble TAA, rapidly released drugs. In addition to instant and sustained release, Tran *et al*. reported an interesting programmable burst release MN using core-shell tip structures with a shell made from PLAG with different degradability outside the drug-loaded water-soluble core 98. Thus, a burst release of the payload was achieved tens of days after injection of MNs, when the shell degraded.

Recently, stimuli-responsive MNs have been reported to facilitate and control payload release to treat skin diseases 99-101. Stimuli-responsive materials comprise a range of compounds capable of responding to changes in their surrounding environment. These systems respond to endogenous stimuli (e.g., pH, redox potential, glucose, and enzymes) and exogenous stimuli (e.g., temperature, electric field, light, and mechanical stress) to release the payload. The spatiotemporal control of the response system enhances the efficacy of drug delivery and minimizes the potential side effects associated with nontarget drug delivery.

Endogenous stimuli-triggered drug release can be achieved by introducing responsive functional groups either in needle tips or encapsulated nanocarriers. In the former case, drugs were directly mixed with polymers before making an MN. For example, Zhang *et al*. developed ROS-responsive MNs by using polymer gels made from poly(vinyl alcohol) and phenylboronic acid crosslinkers as tip materials to deliver drugs for anti-acne therapy 99. After MNs penetrated the skin, the elevated ROS in the skin microenvironment led to a controlled and sustained drug release and effective inhibition of bacterial proliferation. The encapsulation of intelligent nanocarriers in MNs can also offer stimuli-responsive drug release. In one study, Wang *et al*. designed an MN containing pH-sensitive dextran nanocarriers encapsulated with an anti-programmed cell death protein-1 (PD-1) antibody (aPD1), glucose oxidase (GOx), and catalase (CAT), to treat melanoma 100. Thus, the enzyme complex inside nanocarriers generated gluconic acid to promote the gradual self-dissociation of dextran nanocarriers, leading to the sustained release of aPD1 over a three-day administration period.

Exogenous stimulus-triggered drug release is particularly suitable for skin disease, as external therapies, such as light therapy, have been applied to treat skin diseases. As an example, Hao *et al*. integrated NIR-responsive 5-Fu- and indocyanine green (ICG)-loaded monomethoxy-poly(ethylene glycol)-polycaprolactone (MPEG-PCL) nanocarriers (5-Fu-ICG-MPEG-PCL) in an HA MN system as a melanoma therapy 101. 5-Fu-ICG-MPEG-PCL can be delivered transdermally *via* MNs with the release of 5-Fu controlled by NIR irradiation.

### Targeted drug delivery

Targeted delivery systems can prolong drug accumulation in the skin. Moreover, targeted drug delivery is essential for developing precise treatment and reducing toxic side effects. In terms of MNs, the targeting strategies can be classified into three categories: i) delivering targeted drugs such as antibodies or nanocarriers with targeting groups; ii) using MNs themselves to create a specific exogenous targeting site; and iii) controlling tip lengths to facilitate the delivery of drugs to specific skin layers.

Delivery targeting drugs or nanocarriers is the most frequently used strategy in MN-mediated targeted drug delivery. The first investigation combining the approach of nanocarriers with MNs for improved skin drug delivery was reported in 2003 by McAllister *et al*. 102.

**Figure 6:**
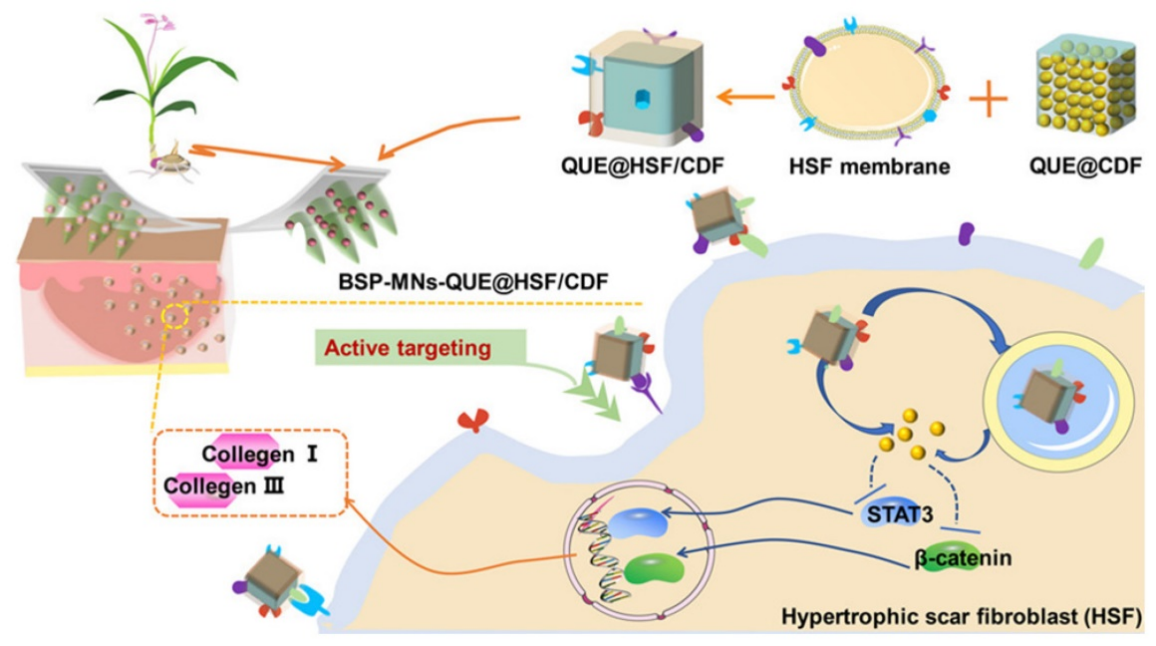
Schematic illustration of fabrication and administration of BSP-MNs-QUE@HSF/CDF for topical anti-hypertrophic scar treatment. Adapted with permission from reference 38. Copyright 2021 American Chemical Society.

In a recent study, Wu* et al.* developed dissolvable *Bletilla striata* polysaccharide (BSP) MNs to deliver hypertrophic scar fibroblast (HSF) membrane-coated nanocarriers for the treatment of hypertrophic scars 38. The HSF membrane of nanocarriers generated homologous targeting effects to dermal fibroblasts and improved the therapeutic effects. In this study, a diphenyl carbonate cross-linked cyclodextrin metal-organic framework (CDF) containing quercetin (QUE) was coated with HSF membrane (QUE@HSF/CDF) and then dispersed in BSP-fabricated dissolvable MNs (BSP-MNs-QUE@HSF/CDF) for local administration. This biomimetic nanodrug delivery system improved efficacy on HSs by regulating Wnt/β-catenin and JAK2/STAT3 pathways and reducing the expression of collagens I and III in HS, and this performance was superior to those of systems without HSF functionalization or the assistance of MNs (**Figure 6**). In another study, Jing *et al*. developed MN delivery of HaCaT cell membrane-coated pH-sensitive micelles for therapeutically active targeting of psoriasis 103. The nanocarriers were internalized by the target cells and accumulated mainly in the active epidermis. This emerging biomimetic targeted delivery strategy is a new approach for improving the treatment of skin diseases.

Many antibody-based therapies have demonstrated good treatment outcomes due to their precise targeting ability. For example, immune checkpoint blockade (ICB) therapy using programmed cell death protein 1 and its ligand (PD-1/PD-L1) and cytotoxic T-lymphocyte-associated antigen 4 (CTLA-4) elicits an antitumor response by inhibiting immune suppressor components 104. Despite their intrinsic targeting ability, the "off-target" effects of antibodies following systemic administration and low response rates remain challenging for ICB therapy. Compared with systemic administration, dermal administration turns out to be a promising route for immunotherapy since skins contain a large population of immune cells that can trigger an immune response. For this reason, the local and targeted administration of ICB formulations using MNs may be a potential solution to maximize treatment outcomes while minimizing side effects. Lan *et al*. investigated MN-mediated delivery of lipid nanocarriers loaded with aPD1 and cisplatin to cancer tissues for squamous cell carcinoma therapy 104. The anti-PD-1/cisplatin NPs delivered through MNs effectively boosted a robust T-cell response by blocking PD-1 in T cells and achieved a synergistic anticancer effect.

Immunotherapy can also be used to treat inflammatory skin diseases through immunosuppression. Bandyopadhyay *et al*. developed an antigen-specific immunosuppressive approach to treat allergic contact dermatitis by topical delivery of hapten and neurokinin-1 receptor (NK1R) antagonists in DMNs 105. This treatment suppressed neuropeptide-mediated skin inflammation in mouse and human skin, promoted deletion of antigen-specific effector T cells, and increased regulatory T cells, which prevented allergic contact dermatitis onset and relapse locally and systemically in an antigen-specific manner.

MNs themselves can serve as exogenous targeting sites for precise treatment. Effective site-specific delivery of the MN technique combined with nanocarriers can be designed and used to reduce the systemic side effects of chemotherapeutics. In a recent study, Chen *et al*. developed a catalytically active MN patch that could locally transform chemotherapeutics from its inactive prodrug form into an active state for the treatment and inhibition of melanoma 106. The MN patches were made from a PVA matrix and contained palladium NPs hosted by TiO_2_ nanosheets (Pd-TNS) as nanofillers. After piercing the skin of the melanoma, the MN patch changed into a swollen and porous hydrogel state. The resulting highly exposed surfaces of Pd-TNSs in the network were in contact with caged *N*-allyloxycarbonyl-caged DOX (alloc-DOX) that diffused into needle tips, thus facilitating the local intertumoral activation of DOX by separation from the cage. This method significantly reduced the side effects of DOX on healthy organs and tissues, thus allowing the increased dose of DOX prodrug for improved treatment outcomes.

Delivering drugs to different layers of the skin may facilitate drug targeting. The design of MN tips with multiple layers of controllable lengths can satisfy this requirement. Yu *et al*. developed a layered MN loaded with the immunosuppressant tacrolimus (TAC) and anti-inflammatory diclofenac (DIC) in different layers of MNs to precisely deliver TAC and DIC to the skin and articular cavity, achieving simultaneous alleviation of psoriatic skin and arthritic joint lesions in psoriasis 107. TAC was loaded into the interlayer of the MNs, while DIC was loaded into the tip layer of the MNs. After MN insertion, the tip layer first pierced the SC and further reached the deeper site of the epidermis. The loaded DIC released from the tip layer diffused into the articular cavity to treat arthritis. The TAC released from the interlayer was retained primarily within the epidermis to treat psoriatic lesions. The MN layer-loaded and site-targeting delivery of TAC and DIC simultaneously alleviated skin psoriasis and joint arthritis and provided a new approach for psoriasis treatment.

### Imaging, diagnosis, and therapeutics

In recent years, the importance of molecular and diagnostic imaging has increased dramatically in treating skin diseases. There are particularly interesting possibilities for combining imaging and therapy within MN-mediated therapy.

Early detection of skin diseases is indispensable for effective treatment. However, fluorescent molecular probes that are capable of doing this are rare. As keloid cells exhibit high levels of fibroblast activation protein-alpha (FAPα) expression, Miao *et al*. designed the FAPa-activatable probe (FNP1), which has a caged NIR dye and a FAPα-cleavable peptide substrate linked by a self-immolating segment 108. FNP1 rapidly and purposefully turns on its fluorescence at 710 nm by a factor of 45 in the presence of FAPα, allowing it to effectively distinguish keloid cells from ordinary skin cells. Combining FNP1 with a simple MN-assisted topical application allows for the sensitive detection of keloid cells in metabolically active human skin tissue. In addition to early detection, the high sensitivity and selectivity of FNP1 will contribute to the development of systematic posttreatment monitoring and assessment methods for abnormal scars.

NIR-II fluorescence bioimaging allows the detection of deep tissue with minimal autofluorescence and tissue scattering 109. Liu *et al*. designed an MN-based NIR-II fluorescent ratiometric sensing system to detect H_2_O_2_ in inflammatory skin *in vivo* 110*.* They developed novel Er^3+^ sensitized upconversion NPs with both excitation (1530 nm) and emission (1180 and 980 nm) located in the NIR-II window. In contact with H_2_O_2_, IR1061 on the surfaces of NPs was degraded in a Fe^2+^-mediated Fenton reaction, leading to the recovery of 980 nm emission from NPs. The emission at 1180 nm remained stable as a reference. Owing to the large anti-Stokes shifting, low autofluorescence, and tissue scattering of the NIR-II upconversion luminescence, inflammation can be assessed dynamically *in vivo* with very high resolution.

With a growing number of MN-mediated imaging and diagnosis methods entering the clinic, various imaging methods will play an important role in facilitating the translation of skin disease therapeutics from bench to bedside.

## The applications of nanocarrier- or MN-based transdermal delivery in treating skin diseases

As most skin diseases originate from the skin, transdermal drug delivery would be a perfect drug administration route for treating abnormal cutaneous lesions. Especially, nanocarrier- or MN-based drug delivery systems have obvious advantages over conventional methods, such as efficient and minimally invasive skin penetration, controllable drug release rates, precise and enhanced targeting, and theranostic functions. The related examples are summarized in the following two tables (**Table 1 and Table 2**), and we introduce their applications in treating psoriasis, dermatitis, melanoma, and other skin diseases in the following section.

### Transdermal delivery for psoriasis treatment

Psoriasis is a common immune-mediated, chronic inflammatory skin disease characterized by sharply demarcated erythematous and scaly skin lesions. It is also associated with undesired cardiovascular, metabolic, and neuropsychiatric effects 167. Psoriasis affects 2-3% of the worldwide population and cannot currently be cured. Traditional systemic agents, such as MTX, cyclosporine, retinoids, and acitretin 168, have poor efficacy, inadequate response, and toxic side effects, such as nausea, fatigue, abdominal pain, diarrhea, headaches, impaired liver or kidney functions, and increased blood pressure 167, 168. Thus, there is a great demand for efficient transdermal drug delivery.

The major manifestations of psoriasis are itchy, scaly, and flaky skin, swelling, and painful and disfiguring lesions 167. Treatments for psoriasis include topical medication and physiotherapy therapy for mild patients and systemic medication for moderate and severe patients 167. Systemic approaches are mainly used in severe conditions or when psoriasis is resistant to topical treatment. If these systemic agents can be locally delivered into the skin, improved therapeutic outcomes and reduced toxic side effects are expected to be achieved. However, the chemical structures of drugs such as MTX determine that they do not have good transdermal efficiency. Only a small proportion of the drug penetrates the skin during transdermal administration. Various treatment strategies have been explored and have achieved good results to address these drawbacks of current topical drug delivery strategies for psoriasis treatment, such as poor transdermal efficiency and ineffective treatment. Therefore, our main objective in this part of the review is to introduce various advanced transdermal approaches, such as nanocarriers and MNs, to effectively treat psoriasis.

#### Nanotechnological approaches in psoriasis therapy

Nanotechnology in psoriasis treatment focuses on the encapsulation of active agents in nano- or micron-sized particles. The encapsulation of the active agent in nanocarriers can improve aesthetics, protect the active agent against degradation, help to target the skin layer, and prolong drug release, providing several other advantages, such as better patient compliance with noninvasive administration, particularly in the case of chronic pathology, and reduced side effects, thereby improving the benefit/risk ratio.

As we introduced in the previous section, enhanced skin penetration is one of the critical issues for treating psoriasis. Liu *et al*. developed liposomal spherical nucleic acids (SNAs) topically applied for psoriasis treatment. The nanocarriers efficiently penetrated the epidermal barriers due to their fluidic nature and specific interaction between lipids and epidermal lipids. Immunological and genetic studies have established that psoriasis pathogenesis is caused by hyperproliferation and disturbed differentiation of epidermal keratinocytes provoked by immune mediators of the IL-23 and IL-17 pathways. With spherical structure, SNAs could efficiently target the gene encoding the mouse IL-17A receptor (IL17ra) and reverse psoriasis's development in imiquimod-treated psoriasis-like mouse skin clinically histologically and transcriptionally 112.

Controlled release of the drugs from nanocarriers also enhances psoriasis treatment outcomes. MTX is the first-line drug for psoriasis treatment. Guo *et al*. introduced the biocompatible aromatic molecule riboflavin into well-defined telodendrimer nanocarriers to enhance the loading capacity of MTX through hydrogen bonding and hydrophobic interactions between riboflavin and MTX 115. As a result, the MTX loading capacity of nanocarriers reached over 20% (w/w) with a particle size of 20-30 nm. The designed interactions in nanocarriers allowed the sustainable release of MTX over 48 h and exhibited a long-lasting efficacy in reducing skin inflammation and excellent hemocompatibility compared with free MTX in psoriasis treatment.

The enhanced targeting ability of nanocarriers can contribute to the improved bioavailability of drugs and psoriasis treatment outcomes. Zhang *et al*. linked HA onto propylene glycol-based ethosomes for topical delivery of curcumin that targeted CD44 in the inflammatory epidermis of psoriatic lesions 79. CD44 protein is highly expressed in the epidermis of inflamed psoriatic skin, while the distribution of HA, a natural ligand for the CD44 protein, is reduced, suggesting that overexpressed CD44 protein could be a potential target for nanocarriers to increase skin drug retention and improve drug efficacy 79. As a result, the *in vivo* psoriatic skin retention of curcumin with HA-ethosomes was 2.3 and 4.0 times that of curcumin ethosomes propylene glycol solution (PGS), respectively. Curcumin has antipsoriasis functions due to its antioxidant activity. The nanocarriers with HA as targeting ligands showed stronger anti-inflammatory effects than the ethosomes and PGS groups.

Nanocarriers can do more than deliver drugs. Some nanocarriers themselves have demonstrated therapeutic effects on psoriasis. It is well recognized that cell-free DNA (cfDNA) plays a critical role in development of psoriasis. cfDNA released from damaged or dead cells binds to LL37, which activates the deterioration of psoriasis. To interfere with DNA-LL37-induced immune activation, Liang *et al*. designed cationic NPs that competitively bound cfDNA and prevented the formation of the DNA-LL37 immunocomplex to treat psoriasis 169. They found that cationic polymers with a high DNA binding affinity effectively pulled cfDNA out of the DNA-LL37 complex, which, in turn, effectively inhibited the activation of plasmacytoid dendritic cells (pDCs) and primary epidermal cells. The results showed that the topical application of cationic NPs on the psoriatic skin of a mouse model greatly reduced scales and erythema, leukocyte infiltration, and proinflammatory cytokines. It is noteworthy that the results were confirmed in a cynomolgus monkey model. In another recent study, Yan *et al*. made poly(2-(dimethylamino) ethyl methacrylate) (PDMA)-grafted silica particles (cSPs) with precisely controlled lengths of cationic hairs for cfDNA scavenging antipsoriasis therapy 170. They found that topically applied cSPs with a size of 700 nm and a PDMA content of 14% displayed the longest retention time in psoriatic skin and exhibited a higher affinity for cfDNA in the dermis. The effective scavenging of cfDNA in the dermis suppressed cfDNA-driven psoriatic inflammation. AuNPs with different structures or components have frequently shown certain therapeutic effects. For example, Han *et al*. devised alkyl-terminated AuNPs (Au_3_@PEG_1000_-octadecyl_30%_) as a topical formulation for psoriasis treatment 86. The NP had a 3 nm gold core grafted with 1000 Da PEG chains, including 30% PEG-octadecyl chains. Interestingly, the application of Au_3_@PEG_1000_-octadecyl_30%_ to psoriatic skin establishing psoriasis was as effective as standard steroid and vitamin D analog therapy. Although the exact mechanism was not clear, it was found that Au_3_@PEG_1000_-octadecyl_30%_ preferentially entered keratinocytes and downregulated the genes that modulate IL-17-enriched signaling pathways, which were associated with epidermal hyperproliferation and inflammation. Furthermore, Au_3_@PEG_1000_-octadecyl_30%_ did not accumulate in major organs and induced long-term toxicity. These examples show that the “skin-nano” interactions may be more complex than we thought and need further investigation.

### MNs in psoriasis therapy

Psoriatic plaques are characterized by thickening of the SC, which can hinder percutaneous drug penetration. MNs can penetrate the skin SC, so the application of MNs to treat psoriasis offers very substantial advantages.

**Figure 7:**
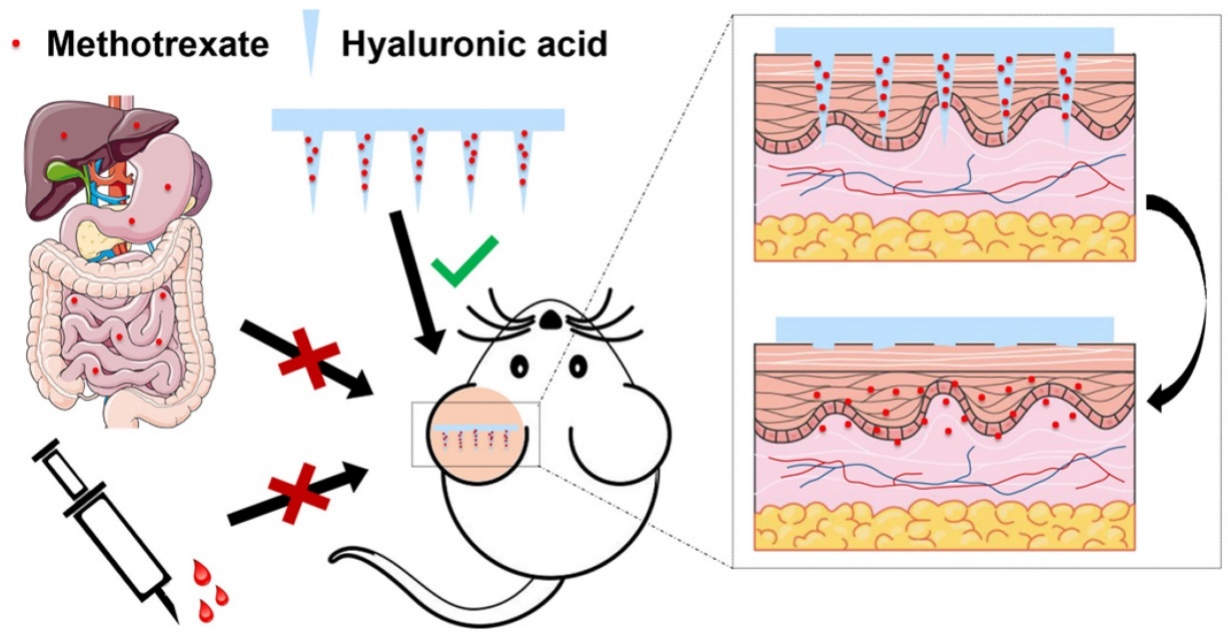
Schematic illustration of an HA-based DMN patch loaded with MTX to improve the treatment of psoriasis. Adapted with permission from reference 94. Copyright 2019 American Chemical Society.

Topical MTX treatment does not have good efficacy due to its hydrophilic nature. Our group developed an MTX-loaded DMN patch made from HA to enhance MTX penetration to treat psoriasis (**Figure 7**) 94. The amount of MTX in each patch could be precisely controlled. Notably, MTX-loaded MNs with sufficient mechanical properties successfully penetrated the imiquimod-induced psoriatic skin of mice and released MTX from DMN tips, thereby alleviating psoriasis-like skin inflammation and reducing skin thickness in mice. In addition, the results from *in vivo* experiments showed that MTX-loaded MNs could achieve similar treatment outcomes as oral administration of MTX but used only half of the drug dose. The MN patch with biocompatible and water-soluble HA as its matrix offered a desirable alternative to conventional oral administration by enhancing the therapeutic effect and demonstrated promising clinical translation potential.

One of the key issues in fabricating drug-loaded MNs is to ensure the uniform distribution of drugs in the polymer matrix, which is beneficial for increasing the drug loading capacity in MNs. However, a high amount of MTX may phase separate with the polymers in needle tips during drying, limiting the drug loading content in MNs. To address this limitation, Tekko *et al*. explored the combination of nanocrystal and DMN technologies as an alternative approach for localized and continuous intradermal administration of MTX 142. Poorly water-soluble MTX nanocrystals with an average particle size of 678 ± 15 nm were produced using a bottom-up technique. The good dispersion of MTX nanocrystals in the polymer matrix led to a drug loading capacity as high as 2.48 mg/array. Moreover, the MTX-loaded DMNs could release MTX in a sustained manner over 72 h. Accordingly, MTX-loaded DMNs could be a promising approach for the effective localized and sustained intradermal delivery of MTX as a potential enhanced treatment for psoriasis.

To increase the targeting of the MN-mediated drug delivery system, Jing *et al*. developed MN delivery of HaCaT cell membrane-coated pH-sensitive micelles for therapeutically active targeting of psoriasis 103. They encapsulated shikonin in HaCaT cell membrane-coated pH-sensitive micelles and discovered that the nanocarriers accumulated mainly in the active epidermis when delivered with MNs. Internalization of the nanocarriers by the target cells led to swelling of the histidine fragments by protonation. Subsequently, it triggered drug release, which increased the therapeutic efficacy of shikonin against imiquimod-induced psoriatic epidermal hyperplasia.

Furthermore, MNs have also been used to deliver antibodies for the precisely targeted treatment of psoriasis. In recent years, early treatment with approved biotherapy has been demonstrated to greatly benefit in improving treatment outcomes and controlling systemic inflammation, suggesting that a similar approach may help optimize the long-term outcomes of psoriasis treatment. It is noteworthy that several biological agents are approved for the treatment of moderate-to-severe psoriasis (e.g., etanercept, adalimumab, infliximab, certolizumab, and ustekinumab) because their targets are central to the pathogenesis of the disease. Among them, anti-tumor necrosis factor-alpha specific antibodies (anti-TNF-α Ab) have been demonstrated to be effective in inhibiting tumor necrosis factor and treating a range of inflammatory diseases. With this premise, Korkmaz *et al*. explored the delivery of anti-TNF-α Ab to the intradermal microenvironment of mice and humans by the DMN 143. In this work, MNs that incorporated antibodies in needle tips were manufactured using a micro-drilling/spin-casting fabrication method. The results demonstrated that MNs effectively delivered anti-TNF-α Ab in the intradermal microenvironment. Significant improvements in psoriatic epidermal proliferation and reduced expression of key biomarkers of inflammation, such as interleukin-1β (IL-1β), supported the effectiveness of MN-mediated antibody delivery strategies.

### Transdermal delivery for AD treatment

AD is a highly prevalent, chronic, relapsing, skin disease that greatly affects the quality of life of patients 171. AD is commonplace in childhood, affecting up to 20% of children and up to 3% of adults in developed countries, with higher estimates in low-income nations 172. From a clinical perspective, patients suffering from AD usually have dry, erythematous, flaky, and cracked skin 171. The origin of AD is uncertain, but both genetic and environmental factors contribute to the disorder's pathogenesis. Although there is no definitive treatment for AD, suppressive medications are designed to control the symptoms of the disease. Yet, the therapeutic outcomes are limited by the poor transdermal delivery efficiency. The current section of the review aims to assess the significance of advanced nanocarrier- or MN-based transdermal approaches toward skin delivery of active ingredients for the effective treatment of AD.

#### Nanotechnological approaches in AD therapy

The currently available drugs have low skin bioavailability and are likely to cause serious adverse events. To address the disadvantages of traditional transdermal strategies associated with the treatment of AD, such as poor stability, complex preparation processes, and skin injury, a broad range of investigations have been carried out to formulate and evaluate nanocarrier-based drug delivery systems 173.

As previously described, enhanced skin penetration is a crucial advantage of using nanocarriers to deliver therapeutics for AD therapy. Kumar and colleagues evaluated the efficacy of piperine-loaded ethosomal creams in treating AD compared to conventional creams 117. The results showed that ethosomal creams displayed higher deposition in the epidermis and dermis and significantly reduced ear and skin thickness, skin severity, white blood cell (WBC), and granulocyte and IgE antibody levels in a mouse model, showing favorable potential for the treatment of AD. In addition, Assem *et al*. prepared micelles containing beclomethasone dipropionate, which were then subjected to dermal deposition tests and morphological examination and eventually formulated as hydroxypropyl methylcellulose hydrogels 118. *In vivo* histopathological studies were performed on both the hydrogel and commercially available ointment Beclozone® to estimate their healing efficiency. The* in vivo* histopathology investigation indicated that the prepared hydrogel successfully treated subchronic dermatitis in animal models in a shorter period than Beclozone®, leading to better patient compliance and fewer side effects. In a recent study, Lee* et al*. formulated liposomal astaxanthin (AST) (L-AST) for AD therapy to address the low bioavailability and solubility of AST 122. The authors compared the anti-inflammatory and anti-dermatological effects of L-AST and free AST in this investigation. The effect of L-AST on an animal model of phthalic anhydride (PA)-induced AD was estimated through analysis of morphological and histopathological changes. The results showed that L-AST penetrated the skin better than free AST, and PA-induced dermatitis severity, epidermal thickening, and infiltration of mast cells in skin tissues were ameliorated by L-AST treatment. In one study, Shadab Md *et al*. designed betamethasone valerate (BMV)-loaded chitosan NPs (BMV-CS-NPs) to improve SC penetration 123. The results showed that BMV-CS-NPs exhibited optimum physicochemical characteristics, including high entrapment efficiency (EE) and loading capacity. The drug permeation efficiency and the amount of BMV retained into the epidermis and the dermis were comparatively higher in the case of BMV-CS-NPs compared to BMV solution. In another study, the pharmaceutical convenience of positively charged nanoemulsions was explored by Hussain* et al*. 124. Hussain *et al*. prepared several batches of nanoemulsions loaded with amphotericin B and assessed both their physical and chemical stability at different pH values and temperatures. The results demonstrated that the positively charged nanoemulsion significantly increased amphotericin B permeation across excised human skin. They proposed that the enhanced drug permeability was attributed to the interaction of the positively charged nanoemulsion with the negatively charged keratinocytes of the SC.

Enhanced targeting of nanocarriers can facilitate the bioavailability of drugs and AD therapeutic efficacy. For example, TAC inhibits the activation of T cells and thus suppresses inflammation. Nevertheless, despite its effectiveness, the most common adverse events with TAC are low and unstable bioavailability, burning, and pruritus at the application site. Pople *et al*. developed a TAC-loaded lipid-NP for the treatment of AD. Compared to the commercial formulation (Protopic®), TAC-loaded lipid-NPs revealed greater TAC penetration across the SC and improved drug retention in the epidermis and dermis. TAC-loaded lipid-NPs also exhibit a greater potential to target dendritic cells and, therefore, to downregulate the spectrum of immune mediators and cytokines involved in AD's pathophysiology 125. TAC-loaded lipid-NPs displayed superior performance, effective skin targeting, and improved safety.

#### MNs in AD therapy

There are few reports of MN-based drug delivery systems for AD therapy due to their excellent transdermal efficiency. For example, TAA is also an efficacious and frequently administered corticosteroid for AD treatment. To improve the skin penetration of TAA, Jang *et al*. designed high-dose TAA-DMNs with a therapeutic dose of TAA (2 mg) encapsulated in a DMN patch 144. In this study, a highly concentrated TAA solution was mixed with HA and polyvinylpyrrolidone (PVP) to make DMNs. The TAA-DMNs with strong mechanical strength were effectively inserted into the skin and displayed adequate anti-inflammatory effects. Notably, a single application of TAA-DMNs was more efficacious than twice-weekly applications of TAA cream. Collectively, this high dose of TAA-DMNs has a beneficial therapeutic effect and a broad application potential in inflammatory skin diseases.

In addition, MNs have also been used to deliver CRISPR-Cas9 to inflammasomes for the precise treatment of AD. Inflammasomes are supramolecular complexes of inflammatory proteins that are responsible for activating the inflammatory response and recognizing pathogens in intrinsic immune development. Among the variegated subtypes of the inflammasome, the nod-like receptor pyrin domain associated protein 3 (NLRP3) is implicated in various inflammatory and autoimmune skin diseases, including psoriasis and AD, and its activation is related to several allergic stimuli in the inflammatory process. Given the possible critical role of the NLRP3 inflammasome in the pathogenesis of AD, Wan *et al*. designed a dissolvable MN that could mediate transdermal delivery of CRISPR-Cas9-based genome-editing agents and dexamethasone for the effective treatment of AD 145. A widely explored genome editing tool, CRISPR-Cas9 was employed as a specific inhibitor to target the NLRP3 inflammasome to ameliorate inflammatory diseases. The disruption of NLRP3 by CRISPR-Cas9-based genome editing inhibits NLRP3 activation at the DNA level, and the destruction of the NLRP3 inflammasome could further improve the sensitivity to glucocorticoid therapy. The results showed that MNs greatly facilitated transdermal delivery of CRISPR-Cas9-based genome-editing agents and dexamethasone, reduced transepidermal water loss, and promoted tissue repair of skin lesions. As expected, MN-assisted genome editing dramatically improved glucocorticoid treatment, outperforming clinically available treatments with dexamethasone cream or TAC ointment.

Although the available drugs for AD are effective, there are some concerns about their safety and side effects. Building MNs with polymers with safer and self-therapeutic functions can solve the problem. Chen *et al*. first designed dissolvable poly-γ-glutamic acid (γ-PGA) MNs with immunomodulatory effects to alleviate AD-like symptoms in Nc/Nga mice 146. Transdermal administration of γ-PGA MNs (γ-PGA MNs) enables the release of γ-PGA into the dendritic cell-rich dermis to interact with dendritic cells, thus modulating immune responses. γ-PGA MNs markedly decreased clinical dermatitis scores, epidermal thickness, and mast cell infiltration in mice by downregulating immunoglobulin (Ig)E and IgG1 levels (Th2-associated antibodies). These findings indicated that γ-PGA MNs may represent an innovative, secure, and reliable therapeutic alternative strategy for AD. In a recent study, Chiu* et al*. reported an epigallocatechin gallate (EGCG)/L-ascorbic acid (AA)-loaded γ-PGA MN that effectively delivers EGCG into the skin to ameliorate AD symptoms 147. Owing to its antioxidant and anti-inflammatory activity, EGCG is a prospective therapeutic agent for the administration of AD. However, the instability inherent in EGCG drastically restricts its bioavailability and clinical efficacy. To achieve antioxidant and immunomodulation, they loaded EGCG and AA-loaded γ-PGA into MNs. MNs protected EGCG not only from oxidation but also as an immunomodulator, downregulating T helper type 2 (Th2)-type immune responses. Once-weekly administration of EGCG-loaded MNs to the AD mouse model for four weeks markedly ameliorated skin lesions and epidermal hyperplasia. Such a delivery strategy maximized the therapeutic effect of synergistic treatment.

In parallel with the revolution in the management and treatment of AD, clinicians have begun to discriminate and better study late-onset AD, which is often more polymorphic, less easily recognized, and deserves a more accurate differential diagnosis. Solutions based on nanocarrier and MN delivery systems appear promising for treating AD, especially recalcitrant dermatitis.

### Transdermal delivery for melanoma treatment

Melanoma is a malignant tumor that originates from melanocytes, accounting for more than 90% of all skin cancer-related deaths, and is characterized by a highly aggressive, metastatic, and poor prognosis 174. Melanomas can be categorized into cutaneous and noncutaneous types 174. In recent years, the incidences of melanoma and mortality rates have been on the rise. As a result, melanoma poses a serious threat to public health and a heavy economic burden. In this section, we introduced examples of using nanocarrier-and MN-based transdermal options for melanoma treatment.

#### Nanotechnological approaches in melanoma therapy

Nanomedicine has gained significant popularity in transdermal drug delivery for melanoma treatment 175, 176. Nanotechnology presented an opportunity to encapsulate anti-melanoma drugs in a nano system, enabling drug targeting to the lesion sites, enhancing transdermal delivery of hydrophobic drugs, achieving controlled drug release, and facilitating more effective combination therapy. The topical drug delivery route is considered an alternative or complementary approach to melanoma treatment because the basal layer of the epidermis, the target site of melanoma treatment, is accessible *via* this route.

**Figure 8:**
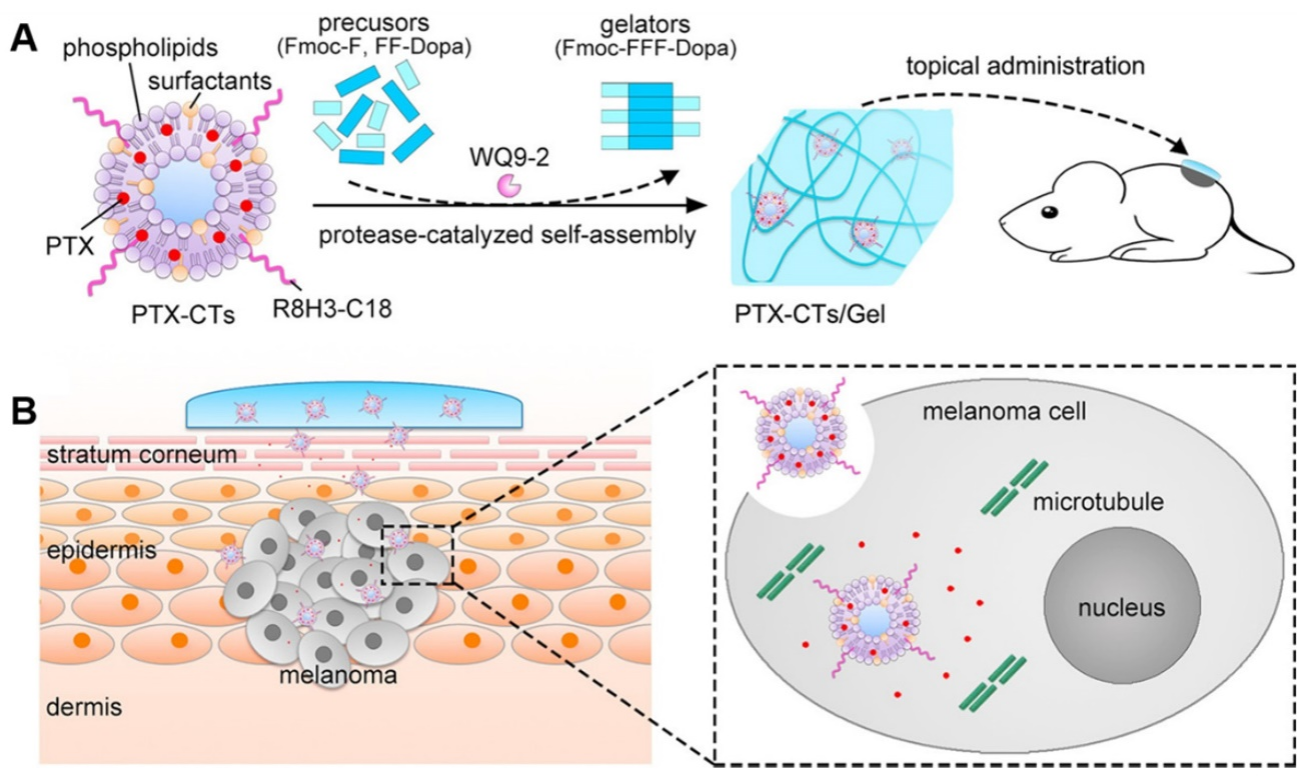
(A) Schematic illustration of the preparation and application of PTX-CTs/Gel as a paintable patch for topical drug delivery. (B) Schematic illustration of enhancement on the transdermal efficiency of PTX by the PTX-CTs/Gel for noninvasive chemotherapy of melanoma. Adapted with permission from reference 126. Copyright 2018 American Chemical Society.

The enhanced skin penetration of nanocarriers has been reported by using some biologically inspired peptides, such as CPPs (cell-penetrating peptides) and TD peptides 177, 126. In one previous study, Jiang *et al.* constructed a paintable oligopeptide hydrogel containing paclitaxel (PTX)-encapsulated and CPP-modified transferosomes (PTX-CTs) to enhance the transdermal delivery of PTX for topical treatment of cutaneous melanoma (**Figure 8**) 126. The PTX-CT embedded hydrogel (PTX-CTs/Gel) served as a patch on the skin of the melanoma tumor that retained PTX-CTs on the skin for an extended period. The deformable PTX-CTs exhibited superior transdermal efficiency with surfactant components and CPP modifications, leading to enhanced tumor penetration and intracellular delivery. PTX-CTs combined with systemic chemotherapy proved to be effective in inhibiting tumor proliferation in a mouse model of B10F16 melanoma. In addition, Zou *et al.* developed a TD-modified vemurafenib-loaded liposome (Vem-TD-Lip) for targeted therapy of melanoma 127. TD can promote penetration through the skin by temporarily opening the intercellular pathway. *In vitro* experiments demonstrated the ability of TD to enhance the transdermal delivery of Vem-TD-Lip. *In vivo* experiments confirmed that Vem-TD-Lip effectively targeted and inhibited melanoma *via* transdermal delivery. The transdermal therapeutic effect of Vem-TD-Lip was superior to that of oral and intravenous injection.

Stimuli-responsive drug delivery systems have shown considerable potential for the efficient local targeting of melanoma treatment. In terms of endogenous stimulus-responsive drug release, Yamazak *et al.* developed a dual-stimuli responsive liposome for controlled transdermal delivery 128. This liposome was modified with pH-sensitive and temperature-sensitive copolymers composed of methoxy diethylene glycol methacrylate (MD), methacrylic acid (MAA), and lauroxy tetra-ethylene glycol methacrylate (LT) (Poly(MD-co-MAA-co-LT)). These copolymer-modified liposomes showed drug release at acidic pH (5~6) and body temperature (35 ~ 37 °C), which corresponded to the endosome environment of melanocytes in the basal layer of the skin. Poly(MD-co-MAA-co-LT)-modified liposomes exhibited good skin penetration. They were efficiently taken up by B16F10 melanoma cells, which released drugs into the endosomes and cytosol of these cells.

In terms of exogenous stimuli-responsive drug release, Wei *et al.* reported thermo-responsive drug-loaded magnetic nanofibers (NFs) coloaded with magnetic NPs (MNPs) and curcumin for local hyperthermic chemotherapy of melanoma 129. The curcumin/MNPs-loaded NFs exhibited an alternating magnetic field (AMF)-responsive heat generation ability. The AMF-induced heating capability of the NFs could achieve the ON-OFF switchable release of curcumin.

Ligand-targeted therapy has great potential in the treatment of melanoma. For example, HA is often used as a promising transdermal targeted delivery ligand due to overexpression of HA receptors on the cell surface in cancerous skin, such as CD44 (cluster determinant 44) and LYVE-1 (lymphatic endothelial HA receptor-1) 178. Beack *et al.* reported a transdermal carbon dot - chlorine e6 - hyaluronate conjugate (Cdot-Ce6-HA) for photodynamic therapy of melanoma 130. Overexpression of HA receptors in tumor tissue enhanced Cdot-Ce6-HA accumulation in melanoma skin. *In vivo* and *in vitro* imaging results confirmed the effective transdermal and intracellular delivery of Cdot-Ce6-HA conjugates to B16F10 melanoma cells in tumor model mice. Wang *et al.* synthesized a cationic polymer (SCP-HA-PAE) by modifying skin/cell-penetrating peptide (SCP) and HA to poly β-amino esters (PAEs) 131. SCP-HA-PAE self-assembled to form micelles (SHPs) for delivering siRNA to skin melanoma. SCP improved the penetration ability of SHP through the skin. HA-specific binding to the CD44 receptor overexpressed in tumor cells enhanced SHP targeting melanoma cells. These advantages exhibited by SHP ensured enhanced siRNA delivery efficiency and effective inhibition of skin melanoma progression. Furthermore, integrin αvβ3, an important cell adhesion molecule, is overexpressed on the cell membranes and perinuclear regions of cancer cells, making it an attractive target for cancer cell targeting. In one study, Gu *et al.* proposed outer membrane vesicles (named TEVs) derived from transformed *Escherichia coli (E. coli)* as carriers for transdermal and tumor-targeted delivery 82. TEVs were functionalized by an αvβ3 integrin targeting peptide and ICG (P-TEVs-G). The lipid fusion and deformable properties of P-TEVs-G led to over 400 μm penetration into the skin and effective targeting of melanoma cells. Similarly, Peng *et al.* used outer membrane vesicles to develop ICG-loaded transdermal nanoplatforms with αvβ3 integrin peptide as a targeting ligand (I-P-OMVs) 175. I-P-OMVs showed excellent SC penetration and superior targeting to melanoma. Upon NIR irradiation, I-P-OMVs not only induced apoptosis in primary tumor cells but also activated TRAIL and delayed the progression, relapse, and metastasis of skin melanoma.

#### MNs in melanoma therapy

Compared to psoriasis or AD, transdermal anti-melanoma therapies demand deeper skin penetration of drugs due to cancer invasion below the skin 179, 180. Therefore, designing MNs with better drug diffusion promotes the melanoma treatment. For the purpose of achieving deeper and faster intradermal payload delivery, Lopez-Ramirez and his colleagues engineered MNs loaded with magnesium microparticles that interacted with water in the skin to produce hydrogen bubbles, providing the essential force for deeper and faster anti-CTLA-4 (a checkpoint inhibitor drug) delivery.^92^. The MNs were applied in an animal model for melanoma treatment. The results indicated that the MNs loaded with magnesium (Mg) microparticles greatly enhanced the delivery of the drugs by lateral and vertical routes. The MNs loaded with Mg microparticles penetrated the skin to a depth of 526 ± 71 μm, while MNs without Mg microparticles penetrated to a depth of 111 ± 79 μm. As expected, in anti-CTLA-4 Ab melanoma treatment, MNs loaded with Mg microparticles delayed tumor growth, achieving superior substantial tumor inhibition and remarkably improved animal survival compared to MNs without Mg microparticles.

Controlled drug release was also achieved by MN-based melanoma therapies. The abnormal characteristics in the tumor microenvironment, such as glucose levels and overexpressed ROS, can design responsive MN drug delivery systems for anti-melanoma therapy. In one study, Duong *et al.* developed an intelligent (pH-responsive) MN delivery system for anti-melanoma therapy in which a nanoengineered DNA vaccine from MNs was assembled with a layer-by-layer coating of ultra-pH-responsive OSM-(PEG-PAEU) and immunostimulatory adjuvant poly(I:C), a synthetic double-stranded RNA 148. Upon skin implantation, the ultra-sharp-pH-responsive OSM-(PEG-PAEU) transformed to anionic copolymers and provoked DNA vaccine and poly(I:C) release owing to electrostatic repulsion. The released adjuvants poly(I:C) and DNA vaccine (ovalbumin, OVA) reinforce the maturation of dendritic cells and inspire type I interferon, thereby triggering the production of antigen-specific antibodies for antibody-dependent cell-mediated cytotoxicity and killing of cancer cells by CD8^+^ T cells. Surprisingly, transdermal application of the smart vaccine formulation evoked a 3-fold higher frequency of anti-OVA IgG1 serum antibodies and 3-fold excess of cytotoxic CD8^+^ T cells in mice compared to the soluble DNA vaccine formulation. In another study, Chen *et al*. constructed an acid- and NIR light-responsive MN system by incorporating CuO_2_ NPs for anti-melanoma treatment 149. In an acidic environment, the encapsulated CuO_2_ NPs could be decomposed into Cu^2+^ and H_2_O_2_. On the one hand, the liberated Cu^2+^ could contribute to the conversion of endogenous H_2_O_2_ to highly toxic •OH for chemodynamic therapy (CDT) by catalyzing the Fenton-like reaction, which could be facilitated by self-supplied H_2_O_2_. On the other hand, the CuO_2_ NPs could effectively dissipate endogenous glutathione (GSH) to enhance ROS-induced tumor cell death. Moreover, the MN system containing CuO_2_ NPs could perform as a photothermal agent to achieve pronounced NIR-mediated heating of melanoma areas in vivo.

**Figure 9:**
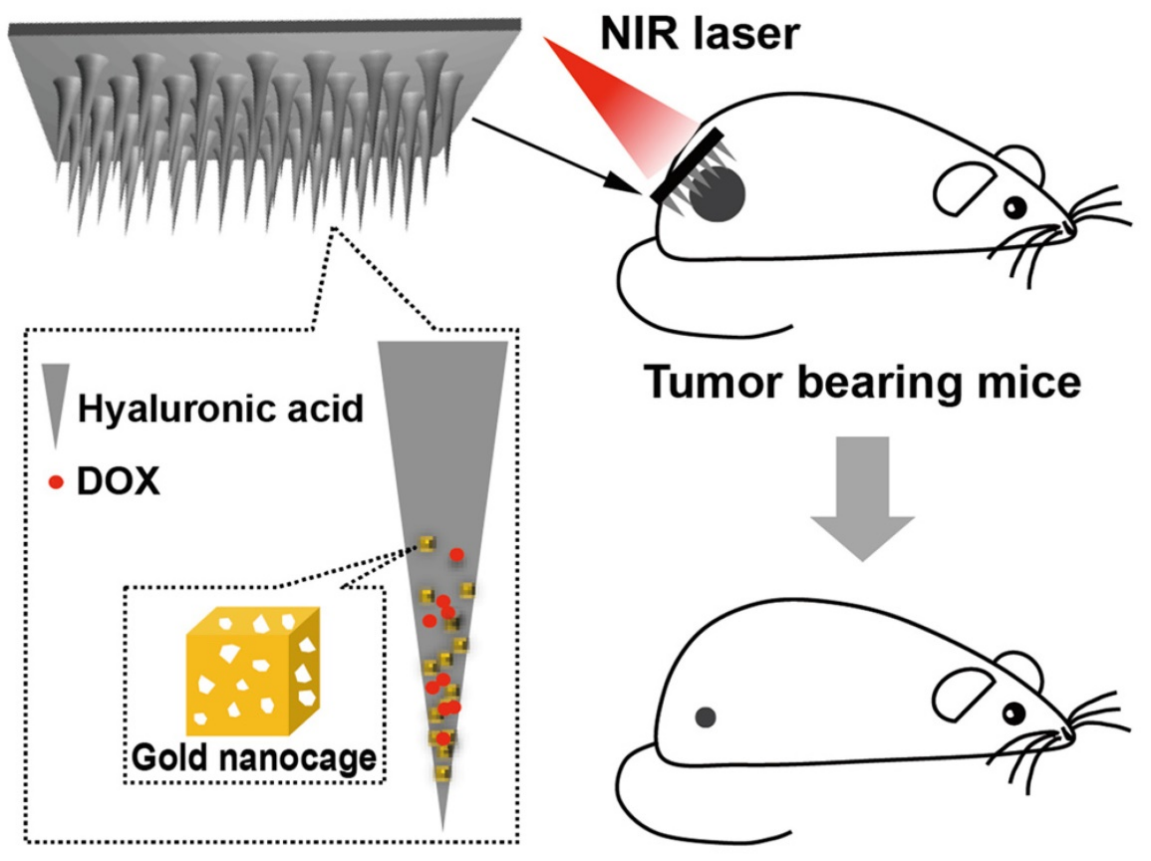
Schematic illustration showing drug/AuNC-loaded dissolving HA DMN system for the combination of chemotherapy and PTT of treating melanoma. Adapted with permission from reference 151. Copyright 2018 American Chemical Society.

Most examples of external stimuli-responsive MNs use light as the trigger. In particular, photothermal effects can be used to trigger drug release or inhibit tumor growth. In one study, Qin *et al*. developed thermal SLN-packaged DMNs as a spatiotemporally controlled pulsatile release system for effective melanoma therapy 150. They encapsulated PTX and the photothermal agent IR-780 in SLNs and then concentrated them at the tips of DMNs (PTX/IR-780 SLNs@DMNs). *In vitro* experiments showed a burst release of PTX/IR-780 SLNs under laser irradiation, followed by a slow release rate that was essentially the same as that of PTX/IR-780 SLNs without laser irradiation, with a cumulative PTX release of approximately 13% at 72 hours. Meanwhile, *in vivo* experiments displayed remarkable antitumor efficacy. In particular, the primary tumor was thoroughly eradicated, with a curable rate of 100% within 30 days and a maximum survival rate of 66.67% after 100 days of treatment. In another study, our group engineered HA-DMNs to deliver DOX-loaded gold nanocages (AuNCs) for the effective and minimally invasive treatment of melanoma (**Figure 9**) 151. After the MNs penetrated the SC barrier of the skin, the needle tips dissolved and released the Dox-loaded AuNCs in the tumor. The photothermal effect of AuNCs triggered by NIR irradiation promoted tumor death and DOX release, which synergistically disrupted the tumors* in vivo*, suggesting a prospective and efficacious drug delivery alternative for melanoma treatment.

MNs can also promote melanoma treatment outcomes with special designs to achieve targeted or precise therapies. Using MNs to deliver immune checkpoint inhibitors is one way to achieve targeted treatment due to their intrinsic targeting ability. ICB therapy elicits an antitumor response by inhibiting immunosuppressive components, including PD-1/PD-L1 and cytotoxic CTLA-4. Ye *et al*. developed a synergistic immunotherapy strategy to treat melanoma by locally targeting the immunoinhibitory receptor PD-1 and immunosuppressive enzyme indoleamine 2,3-dioxygenase (IDO) through an MN-based transdermal delivery method 152. The embedded immunotherapeutic nanocapsule loaded with aPD1 is assembled from HA modified with 1-methyl-DL-tryptophan (1-MT), an inhibitor of IDO. By using the B16F10 mouse melanoma model, they proved that the synergistic treatment achieved vigorous antitumor efficacy, which was associated with enhanced effective T-cell immunity and reduced immunosuppression at local sites. In another study, Ye *et al*. designed a B16F10 melanoma vaccine patch that directly targets antigen-presenting cells (APCs) through transdermal delivery of tumor lysates and melanin 153. Upon insertion into the skin, the MN gradually released the inactive whole tumor lysate, facilitating dendritic cell (DC) uptake and presentation of antigen, which in turn promoted immune activation. Simultaneously, the presence of melanin, a natural pigment available throughout the tumor lysate, enabled local temperature increase through NIR laser-mediated photothermal effect, which further contributed to the enhanced uptake of tumor antigens by dendritic cells and antitumor immune response. The *in vitro* results revealed that the strategy enhanced the effect of the transdermal vaccine, delayed the growth of distant tumors, and improved long-term survival.

Cell therapies are also considered precise treatment strategies that benefit from MN delivery methods. Recently, Chang *et al*. first designed a cryogenic MN patch (CryoMN) for the transdermal delivery of living cells to treat subcutaneous melanoma tumors 154. The CryoMNs were manufactured by progressive cryogenic micromolding of the optimized cryogenic medium with suspended OVA-pulsed dendritic cells. CryoMNs could maintain the viability and proliferative ability of OVA-pulsed dendritic cells and had enough mechanical strength to penetrate the skin to deliver the cells, which evoked a more robust antigen-specific immune response and enhanced the tumor inhibition effect compared to intravenous and subcutaneous injections of the cells.

There are particularly interesting possibilities for combining imaging and therapy within MN-mediated therapy. Due to the superior photostability and photothermal conversion efficiency of aggregation-induced emission luminophores (AIEgens), PTT based on AIEgens is promising for superficial tumor therapy. Recently, Wei *et al*. developed a DMN system to topically deliver NIR950 (AIEgen)-loaded polymeric micelles (NIR950@PMs) for the treatment of melanoma 155. In this study, the NIR950@PMs were concentrated at the tips of the MNs. With the strong anti-photobleaching properties of NIR950, high-resolution *in vivo* optoacoustic imaging was achieved, which could be employed to monitor the distribution of NIR950 in real-time and precisely regulate laser irradiation for the complete removal of tumor lesions.

In addition to treatment, MNs can also assist in the early diagnosis of melanoma, which has the potential to reduce the mortality of the disease. Ciui *et al*. designed a new fully integrated wearable bandage and MN electrochemical sensing platforms to detect tyrosinase on the skin surface and subcutaneously, respectively, for potential melanoma screening 156. This wearable sensor minimizes the need for biopsies in doctors' offices and clinics and the associated delays and anxiety, thus bringing new possibilities for point-of-care cancer screening devices.

### Transdermal delivery for hemangioma treatment

Infantile hemangiomas are the most common soft-tissue tumors in infancy, with a prevalence of 4.5%. They appear shortly after birth, grow to peak size by approximately nine months of age, and then involute over months to years 181. Local medication is suitable for superficial infantile hemangiomas. Beta-blockers (e.g., propranolol ointment, timolol cream, *etc*.) 182, glucocorticoids, bleomycin, rapamycin, and other antitumor drugs can be employed.

#### Nanotechnological approaches in hemangioma therapy

To enhance skin penetration of propranolol, Khalil *et al*. designed lecithin/chitosan NP-loaded propranolol for the treatment of infantile hemangiomas 132. They topically applied hydrogels containing propranolol-loaded lecithin/chitosan NPs to rats and found that skin deposition in rats showed a 1.56-1.91-fold higher accumulation of propranolol from lecithin/chitosan nanocarriers compared to the drug solution.

The enhanced targeting ability of nanocarriers can contribute to the improved bioavailability of drugs and hemangioma treatment outcomes. Guo *et al*. encapsulated propranolol-loaded and CD133 aptamer-conjugated liposomes in PLGA microspheres (PCLIMs) for sustained and targeted treatment of hemangiomas 133. The results revealed that PCLIMs displayed optimal efficacy against hemangiomas in nude mouse hemangioma xenografts, as reflected by a remarkable decrease in hemangioma volume, weight, and microvessel density (MVD). Importantly, propranolol-loaded CD133 aptamer-conjugated liposomes released from the microspheres enabled targeted killing activity against CD133-positive HemSCs. Thus, this study indicated that PCLIM achieved sustained and targeted hemangioma treatment, resulting in measurable suppression of hemangioma.

#### MNs in hemangioma therapy

**Figure 10:**
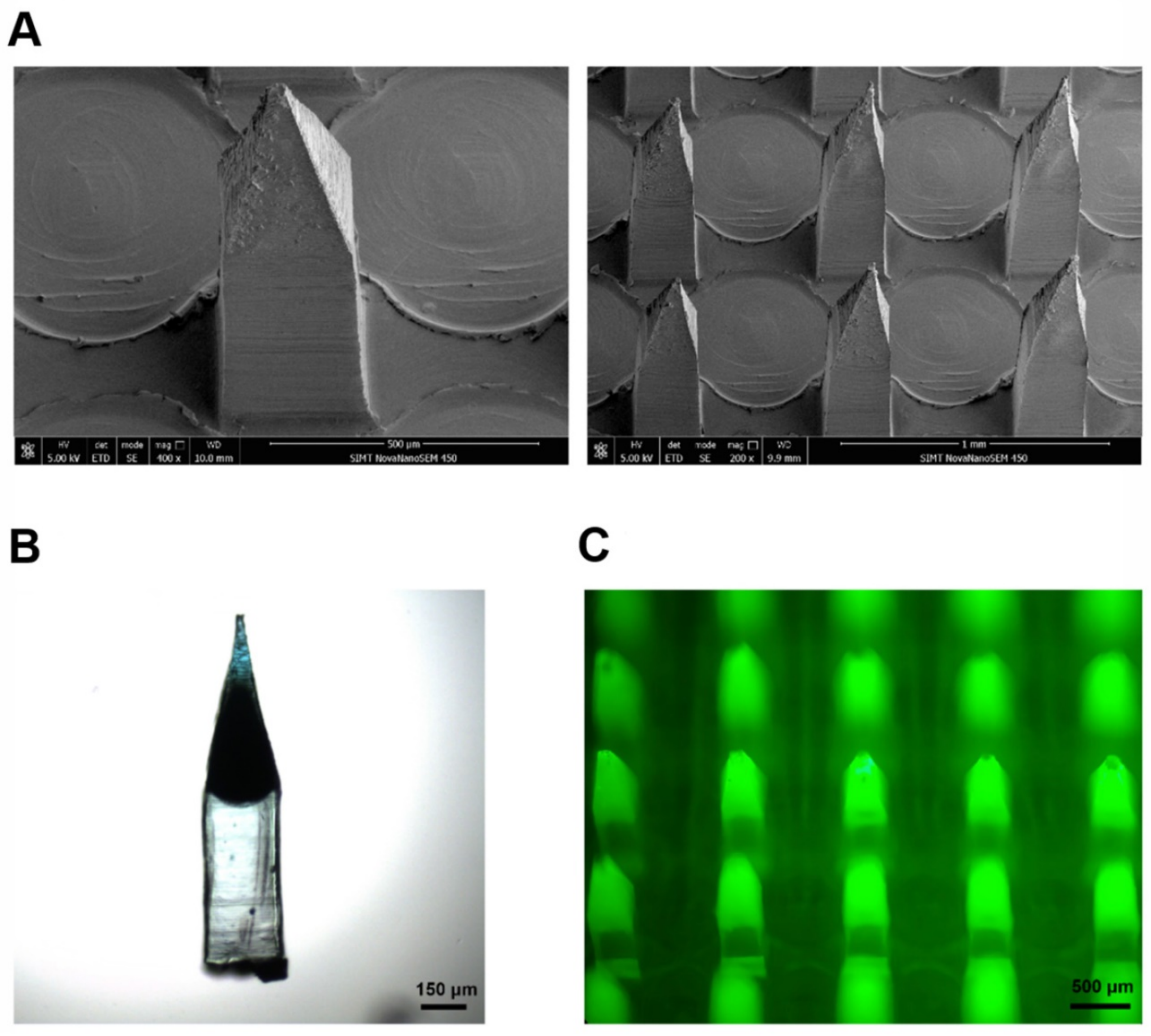
Characterization of the MNs. (A) Scanning electron microscopy images showing the morphology of the MNs. (B) Drug distribution in the tip of the needle was illustrated by methylene blue. (C) Fluorescence microscopy images of the MNs with fluorescein isothiocyanate isomer (FITC) as a tracer reagent. Adapted with permission from reference 158. Copyright 2021 Multidisciplinary Digital Publishing Institute.

Although transdermal treatment of infantile hemangiomas with drug-loaded nanocarriers has been described, their transdermal efficiency is still restricted. Applying MN-delivered drugs to treat hemangiomas might become a revolutionary therapeutic option. In a recent study, He *et al*. fabricated DMNs by a two-step casting procedure that used HA and PVP as matrix materials for loading propranolol hydrochloride in needle tips (**Figure 10**) 158. The MNs had outstanding mechanical strength to penetrate SC, as well as superior dissolution ability to release propranolol hydrochloride. DMNs dramatically enhanced the penetration and skin retention of propranolol compared to solutions.

### Transdermal delivery for alopecia treatment

Androgenetic alopecia (AGA), also known as male-pattern baldness, is one of the most prevalent chronic disorders in dermatology caused by the progressive loss and thinning of scalp hair and affects hundreds of millions of adult men 183. Currently, most treatments for hair loss consist of medication or hair transplantation. However, both approaches have limited effects on regenerating new HFs. The Food and Drug Administration (FDA) has approved two medications for the treatment of AGA, including topical minoxidil and oral finasteride. In this part of the review, we summarize and outline the current advanced transdermal strategies for treating alopecia based on nanocarriers and MNs.

#### Nanotechnological approaches in alopecia therapy

As described, enhanced skin penetration is also crucial for AGA treatment. In the study by Wilson* et al*., ethosomes were used as drug carriers to deliver finasteride to HFs 134. The objectives of this investigation included reducing the amount of finasteride that might reach the systemic circulation by enhancing the targeting of finasteride to the pilosebaceous units of HFs. The outcomes demonstrated that spherical ethosomes exhibited excellent permeability to rat skin and the frontal scalp skin of human cadavers compared to unencapsulated finasteride.

**Figure 11:**
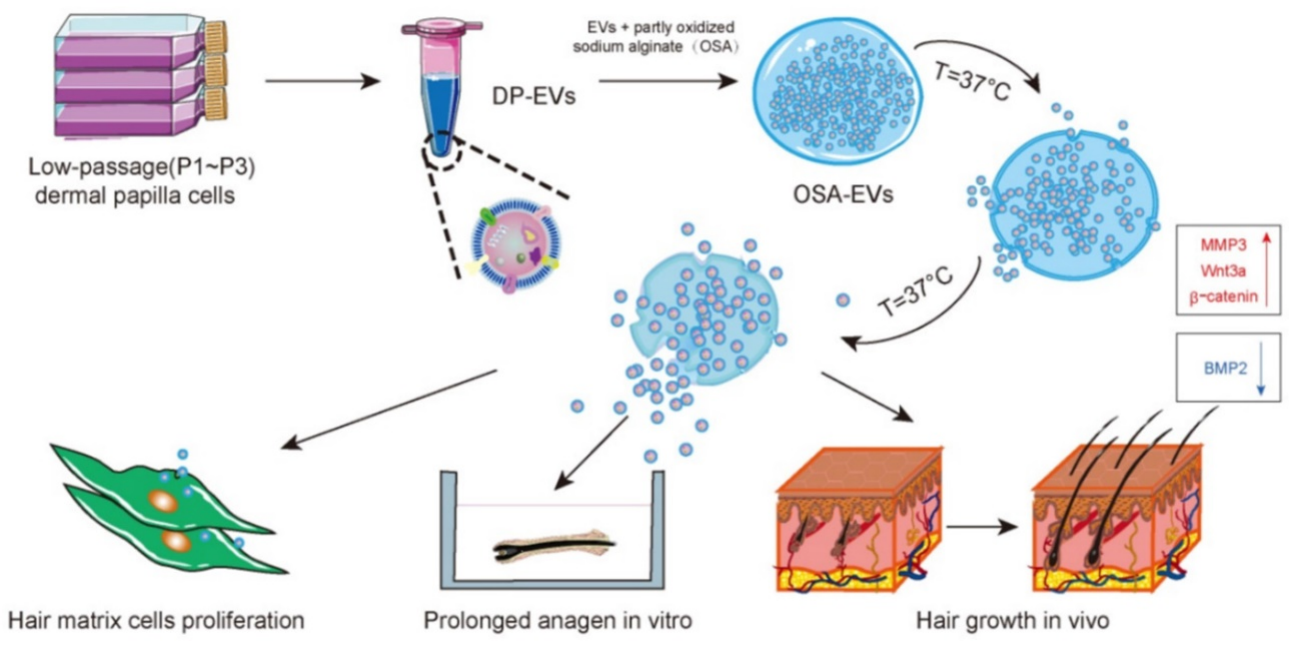
Schematic illustration of the preparation and functional mechanism of OSA-EV nanospheres. Adapted with permission from reference 136. Copyright 2020 Ivyspring International Publisher.

Controlled release of the drugs from nanocarriers also enhances AGA treatment outcomes. Dermal papilla (DP) cells are specialized stem cell niches of mesenchymal origin and are widely considered central to triggering hair recycling through paracrine signaling mechanisms. DP cell-derived extracellular vesicles (DP-EVs) may have a critical function in hair regeneration. However, the instability of EVs *in vivo* and the short retention after transplantation hinder their clinical applications. Chen and his colleagues encapsulated DP-EVs in oxidized sodium alginate (OSA) hydrogels (OSA-EVs) as a sustained-release system to enhance the potential therapeutic effects of DP-EVs (**Figure 11**) 136. They found that DP-EVs were gradually released as the hydrogel degraded. The hydrogel significantly improved the stability of vesicular proteins and retention of EVs *in vitro* and *in vivo*. The OSA-EVs contributed to the proliferation of hair stromal cells, prolonged the anagen phase of cultured human hairs, and accelerated the regrowth of back hairs after hair removal in mice. These effects may be attributed to the upregulation of hair growth-promoting signaling molecules (e.g., Wnt3a and β-catenin) and the downregulation of the inhibitory molecule BMP2. This study is a significant step forward in the exploration of DP-EVs for hair loss, and it may be a substantial advancement in the long-term therapeutic use of EVs.

#### MNs in alopecia therapy

The greatest challenge with topical formulations in the treatment of AGA is that the site of action of HFs is primarily located beneath the SC 40. It is anticipated that drugs must be transported to the deeper skin layers where HFs are located for the treatment of AGA. One study by Cao *et al*. designed an MN system with finasteride nanostructured lipid carriers (FIN-NLC-MNs) for the targeted delivery of finasteride to HFs 160. The FIN-NLC-MNs could deliver bioinspired lipid nanocarriers deep into the skin, and FIN-NLC further specifically delivered finasteride to the HFs. The mechanism of FIN-NLC-MN treatment includes upregulation of hair growth-promoting signals such as β-catenin, IGF-1, and vascular endothelial growth factor (VEGF) while downregulating inhibitory signals such as SRD5A2, transforming growth factor-β (TGF-β1), and IGF-1. *In vitro* and *in vivo* permeation studies demonstrated that FIN-NLCs enhanced finasteride permeation and retention, primarily through the HF pathway, reversing the miniaturization of HFs and promoting hair growth in AGA.

**Figure 12:**
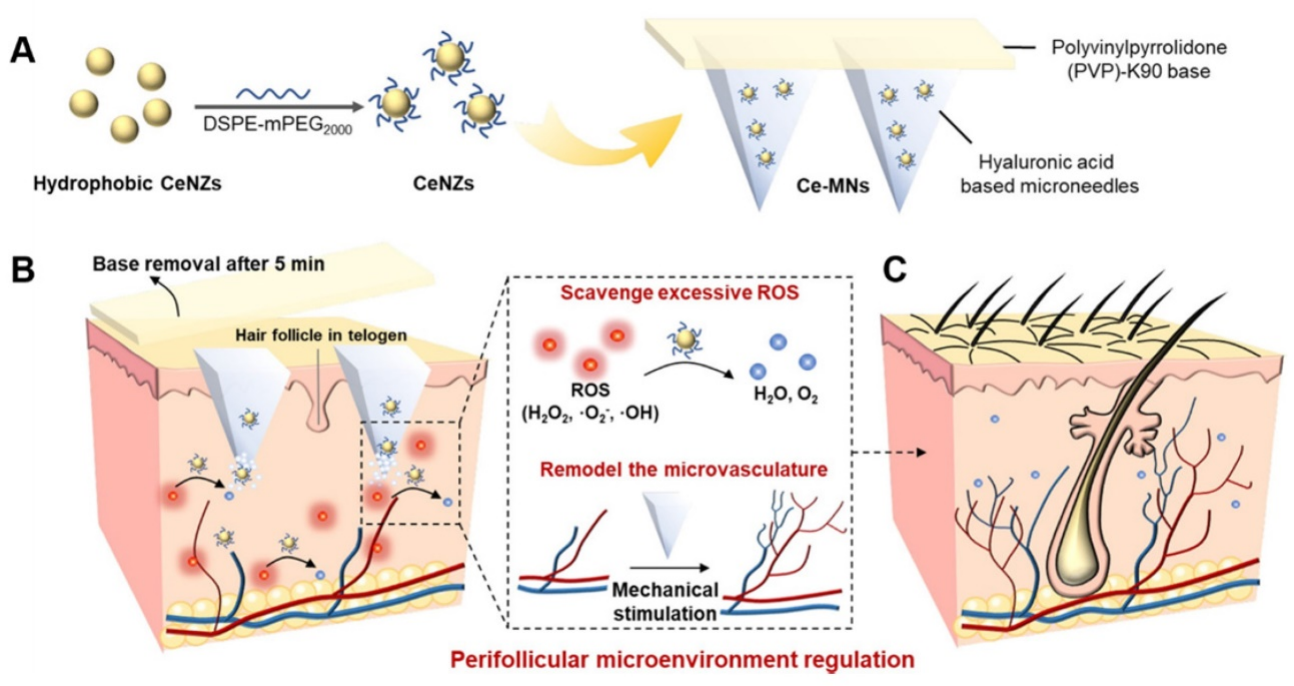
Schematic illustration of AGA therapy through a ceria nanozyme (CeNZ)-integrated MNs (Ce-MNs) patch. (A) Fabrication of Ce-MNs. After modification by DSPE-mPEG_2000_, the CeNZs are encapsulated in the HA-based MNs. The backing layer of the patch is made of PVP-K90. (B) Five minutes after the Ce-MNs are applied to the skin, the PVP patch backing could be detached from the MNs. CeNZs can be delivered into the dermis and epidermis directly to scavenge excessive ROS. The mechanical stimulation induced by the administration of Ce-MNs can remodel the microvasculature in the perifollicular microenvironment. (C) Subsequently, the hostile oxidative microenvironment around the hair follicles is reshaped and angiogenesis is promoted by Ce-MNs, resulting in a fast onset of telogen-to-anagen transition of hair follicles. Adapted with permission from reference 161. Copyright 2021 American Chemical Society.

Another recent paper by Yuan *et al*. engineered a ceria nanozyme (CeNZ)-integrated MN patch (Ce-MNs) that can simultaneously reduce oxidative stress and facilitate angiogenesis to reshape the perifollicular microenvironment for AGA treatment (**Figure 12**) 161. Based on the favorable mechanical strength of CE-MNs, the encapsulated CeNZs with catalytic and superoxide-mimetic activities could be transported efficiently into the skin to scavenge excessive ROS. In addition, the mechanical stimulation evoked by MN administration could remodel the microvasculature in the balding area. In the AGA mouse model, Ce-MNs manifested accelerated hair regrowth at a low dosing frequency without inducing apparent skin damage. Thus, Ce-MNs with perifollicular microenvironment-shaping functions displayed enormous potential for the clinical treatment of AGA.

### Transdermal delivery for facial pigmentation (melasma) treatment

Melasma, the most common facial pigmentation disorder, is a chronic and relapsing disease characterized by irregular brownish macules or patches that are symmetrically distributed on photoexposed areas 184.

#### Nanotechnological approaches in melasma therapy

Enhanced skin penetration of the nanocarrier can improve the effectiveness of melasma treatment. Li *et al*. formulated ascorbyl palmitate encapsulated transfersomes to treat melasma 137. Ascorbic palmitate is extensively used in topical medications or cosmetic formulations for melasma treatment. In this study, the deformable transfersomes squeezed through skin pores in response to transepidermal hydration gradient forces, resulting in a 14.1-fold increase in ascorbic palmitate accumulation into the epidermis. In addition, in a rat melasma model, ascorbic palmitate-loaded transfersomes exhibited superior anti-melasma efficacy to free ascorbic palmitate, effectively alleviating oxidative stress and inflammation in the skin.

#### MNs in melasma therapy

Recently, transdermal delivery of tranexamic acid using biocompatible polymer MNs has provided an effective method for treating melasma. Xing *et al*. engineered a DMN patch containing tranexamic acid to treat melasma 163. Tranexamic acid has been utilized in the management of melasma 185. It is believed to act in multiple pathways, including preventing triggers from activating melanocytes by inhibiting the plasminogen activator system in epidermal basal cells and keratinocytes. The pharmacokinetic investigation revealed that the bioavailability of tranexamic acid from DMNs was more than 1.3 times higher than oral administration. In pharmacodynamic studies, the tranexamic acid DMN significantly reduced melanin deposition in the skin of melasma guinea pigs after eight consecutive doses. The study demonstrated that tranexamic acid MNs could maintain good drug stability under various conditions, including high temperature, high humidity, and light irradiation.

### Transdermal delivery for scar treatment

The scar is a collective term for the morphology, appearance, and histopathological changes of normal skin tissue caused by different traumas. It is typically categorized into keloids and hypertrophic scars, which are histologically characterized by chronic inflammation and excessive extracellular matrix (ECM) deposition 186. Currently, surgical excision, laser treatment, steroid injection, and silicone gel treatment are commonly employed in clinical scar treatment 187. Although certain efficacy has been achieved, there are still some shortcomings, such as the high cost of treatment and long treatment period, which bring economic, physical, and mental burdens to patients. For example, steroids are a conservative treatment that typically takes several years to treat even a single, small lesion 188. Moreover, the risk of recurrence is high, and additional long-term treatment is essential.

#### Nanotechnological approaches in scar therapy

Enhanced skin penetration from nanocarriers can increase scar treatment outcomes. Chen *et al*. examined the effect of papain-loaded elastic liposomes (PELs) on hyperplastic scars by topical application 139. They found that PEL had higher cumulative amounts and fluxes in the skin and displayed higher drug deposition than papain solution. After topical application of PEL, hypertrophic scars in rabbit ears were inhibited. In addition, thickening of the dermal layer of the scars, decreased microvessels, reduced collagen fibers with the regular arrangement, and regulation of TGF-β_1_/Smad and NF-κB signaling pathways were observed.

The enhanced targeting ability of nanocarriers facilitates the utilization of the drugs and the effectiveness of scar treatment. Theoretically, ECM deposition relies on the balance between matrix metalloproteinases (MMPs) and tissue inhibitors of metalloproteinases (TIMPs). TIMP-1 is associated with ECM degradation and therefore may be a prospective and promising therapeutic target. Interfering with TIMP-1 expression using siRNA efficiency is a promising strategy for scar treatment. In a recent study, Aoki *et al*. developed supercarbonate apatite (sCA) NPs that encapsulated TIMP-1 small interfering RNA (siTIMP1) 140. Apatite, a constituent of vertebrate tissues with a nonimmunogenic nature, could address the immunogenicity-related safety issues of vectors for siRNA delivery. This study showed that scar formation, scar cross-sectional areas, collagen densities, and type I and type III collagen levels were significantly decreased at the lesion site after sCA-siTIMP1 injection. Accordingly, sCA-siRNA delivery may be an effective approach for keloid treatment.

#### MNs in scar therapy

Enhanced skin penetration from MNs can promote scar treatment. Our group exploited bleomycin-loaded HA-DMNs (BMNs) to improve the treatment of hyperplastic scars painlessly and efficiently 165. Bleomycin is a glycopeptide antibiotic that has been proven to be efficacious in the management of keloids and hyperplastic scars. The main mechanism of action of bleomycin is the scissoring of the DNA strand, where bleomycin binds to the DNA by electrostatic attraction and breaks the DNA backbone, ultimately leading to cell cycle arrest 165. The results showed that our BMNs had sufficient mechanical strength to pierce the skin with their needles dissolved within 10 min to release 46% bleomycin. In addition, BMNs inhibited the proliferation of human HSF and the secretion of TGF-β1 *in vitro*. Therefore, BMNs provide a potential route to treat hypertrophic scars in a convenient, efficient and minimally invasive manner.

## Future perspectives and conclusions

Following an in-depth review of the available contents and therapeutic evidence, we have recognized that significant advances have been made in using different advanced drug delivery strategies to treat dermatological conditions. These advantages include improving the transdermal efficiency and bioavailability of drugs and enabling precise diagnosis and treatment of diseases. Furthermore, stimuli-responsive drug release and tissue-dependent or cell-specific targeting strategies enabled more precise therapies at the molecular level, thus broadening the pathway to personalized therapy.

While several studies have been performed to address the pharmacological and therapeutic feasibility of the advanced transdermal delivery system in managing various skin diseases, many important fundamental questions still need to be addressed to promote the clinical translation of laboratory inventions. Compared with MNs, the transdermal efficiency of nanocarrier-based drug delivery is less due to the complete non-invasive nature of nanocarriers. However, this can be compensated by increasing the amount of drugs topically applied on skins, which can't be achieved by MNs with a limited volume of needle tips. Moreover, a profound understanding of skin-nano interaction will further advance the design of nanocarriers for transdermal applications. The nanocarriers integrated with imaging functions or special chemical labels can assist the study of their skin entry mechanism, skin retention time, targeting ability, and dermal fate, making it easier for researchers to evaluate therapeutic results and optimize the formations 16, 49, 85, 189. Especially, developing nanocarriers with special-designed responsive imaging signals will be beneficial for studying the dynamic processes of nanocarriers in the skin, such as drug release from nanocarriers. More importantly, it is important to improve our knowledge of the interaction between nanocarriers and diseased skin lesions. For example, the penetration of nanocarriers in specific disorder skin lesions, such as psoriasis and AD, can be higher, as barrier-disrupted SC increases the permeability of nanocarriers 190. Furthermore, much effort must be made to evaluate and optimize chemical or physical parameters of nanocarriers, such as sizes, surface chemistry, and stability, to further improve their therapeutic efficacy for skin diseases. Based on our investigation, the principles that influence the skin permeability of nanocarriers depend on the combined effects of chemical and physical properties, which may vary between different nanoformulations. For example, the impact of the surface charge of nanocarriers on skin permeation remains controversial 55, 63. Therefore, the nano-skin interactions of different nanocarriers may require an independent study. Furthermore, we should choose the most suitable nanoformulations based on the pathophysiological differences of diseases. For example, nanocarriers that enter the skin through HFs might be better for treating AGA or acne vulgaris 134. As another example, liposomes will rapture and fuse with the cell membrane of SC, thus limiting the penetration depth. Yet, this unstable property might be suitable for treating AD by retaining drugs in the epidermis 191. Moreover, the development of disease-specific nanocarriers can potentially enhance treatment outcomes. The fate and pharmacokinetics of topically applied nanocarriers are particularly interesting, which impacts the therapeutic efficiency. In addition, the permeability and intracellular behavior of nanocarriers must be studied at an advanced level to reveal the targeting or treatment mechanism at the molecular level and reduce any potential toxic side effects.

In terms of MN-based drug delivery, efficient drug loading methods, intelligent drug release mechanisms, and pharmacokinetics should be carefully studied. The skin penetration efficiency of the MN-based delivery method should be higher than the nanocarrier-based method. However, as we mentioned, the limited drug loading content is a great challenge for MNs. For polymer MNs, developing strategies to uniformly disperse drugs within polymer MNs can be one way to improve drug loading content 142, as the separation between drugs and polymers may lead to the fracture of needle tips. Yet, considering the limited volume of MNs, improving the efficacy of loaded drugs is also important. The delivery of more effective bioagents, such as antibodies, for skin disease treatment, is a promising option. Combining functional nanocarriers and MN for dermatological therapy and diagnosis can also potentially increase therapeutic outcomes. The diverse chemical designs and physical properties of MN tips and encapsulated nanomaterials can achieve better control of pharmacokinetics and provide more treatment options 96, 100. Currently, the reported literature regarding using responsive MN-based drug delivery systems for skin diseases treatment has focused on melanoma treatment. There is an urgent demand for developing stimuli-responsive MN-based transdermal delivery strategies for other skin diseases, which can be even more intelligent by using diagnostic MNs to guide the drug release and achieve personalized therapy 192.

In addition to the fundamental questions, we should address the issues in the applications. As shown in** Table 3** and **Table 4**, several investigations using nanocarriers and MNs have been approved for clinical trials to treat diverse skin diseases, suggesting the emergence of nanocarriers and MNs have led to satisfactory therapeutic outcomes. Yet, more efforts should be made to push forward the clinical translation of such a drug delivery system. For example, some transdermal technologies require complex manufacturing processes, which have difficulties in mass production. Significantly, the incorporation of NPs into MNs increases the complexity of the MN manufacturing process, which is considered one of the major barriers to the clinical translation of MNs. Therefore, it is important to develop low-cost, reliable means of mass production of nanocarrier- or MN-based formulations to ensure their successful application. More importantly, the safety of MNs and NPs should be thoroughly characterized to reduce the long-term toxicity of these drug delivery systems. Furthermore, to make the various advanced transdermal strategies mentioned in this review have a real clinical impact, clinicians, especially dermatologists, should be well informed about such technologies and participate in the product development process with scientists from other fields. Moreover, regulations regarding the clinical translations and applications of various nanocarrier- or MN-based transdermal strategies should be further discussed and determined.

Currently, many people sustain damage from a variety of dermatological conditions, and it is crucial to explore efficacious therapies to achieve the desired outcomes. Advanced drug delivery strategies based on nanocarriers or MNs are at the forefront of clinical research and are used to revolutionize the treatment of skin diseases. It is hoped that the development of advanced transdermal delivery technologies based on those mentioned in the text will contribute to improved disease detection, diagnosis, and management while enhancing the health-related quality of life of patients with skin diseases worldwide.

## Figures and Tables

**Table 1:** Nanotechnological approaches in skin disease therapy.

	Nanocarriers		Applications	References
	Type & Features	Loading Drugs		
**Psoriasis**	Flexible liposomes composed of phospholipids, Tween 80, and sodium cholate	Trans-retinoic acid & betamethasone	Enhanced penetration	111
	Liposomal spherical nucleic acids	-	Enhanced penetration	112
	Penetration enhancer containing vesicles based on nanogel	MTX	Enhanced penetration	113
	Liquid crystalline nanoparticulates	Berberine oleate	Enhanced penetration	114
	Telodendrimer nanocarrier	MTX	Controlled release	115
	HA-modified ethosomes	Curcumin	Targeted delivery	79
	Mannosylation-modified liposomes	Celastrol	Targeted delivery	116
**AD**	Ethosomal creams	Piperine	Enhanced penetration	117
	Hydroxypropyl methylcellulose hydrogels	Beclomethasone	Enhanced penetration	118
	Eutectic oil-based microemulsions	TAC	Enhanced penetration	119
	Nanoemulsions	α-tocopherol & γ-tocotrienol	Enhanced penetration	120
	PLGA nanocarriers	Dictamnine	Enhanced penetration	121
	Phosphatidylcholine liposomes	AST	Enhanced penetration	122
	Chitosan NPs	BMV	Enhanced penetration	123
	Positively charged nanoemulsions	Amphotericin B	Enhanced penetration	124
	Dendritic cells targeting lipid- nanocarriers	TAC	Targeted delivery	125
**Melanoma**	CPP-modified transfersomes	PTX	Enhanced penetration	126
	TD-modified liposomes	Vemurafenib	Enhanced penetration	127
	pH and temperature dual-sensitive liposomes	Calcein	Controlled release	128
	Thermo-responsive nanofibers loaded with magnetic NPs	Curcumin	Controlled release	129
	HA-modified carbon dots	Chlorin e6	Targeted delivery	130
	Skin/cell-penetrating peptide (SCP)- and HA-modified micelles	siRNA	Targeted delivery	131
**Hemangioma**	Lecithin/chitosan-based NPs	Propranolol	Enhanced penetration	132
	CD133 aptamer-conjugated liposome-microspheres	Propranolol	Targeted delivery	133
**Alopecia**	Ethosomes	Finasteride	Enhanced penetration	134
	Microemulsions	Finasteride	Enhanced penetration	135
	DP cells derived EV	-	Controlled release	136
**Melasma**	Transfersomes	Ascorbyl palmitate	Enhanced penetration	137
	Multilayered vesicles aspasomes	Mg ascorbyl phosphate	Enhanced penetration	138
**Scar**	Elastic liposomes	Papain	Enhanced penetration	139
	Super carbonate apatite NPs	TIMP-1 small Interfering RNA	Targeted delivery	140

CPP: cell-penetrating peptide; SCP: skin/cell-penetrating peptide.

**Table 2:** MNs approaches in skin disease therapy.

	MNs		Applications	References
	Type & Features	Loading Drugs		
**Psoriasis**	HA MNs	MTX	Enhanced penetration	94
	PLA MNs	Calcipotriol	Enhanced penetration	141
	PVP/PVA MNs	MTX nanocrystal	Controlled release	142
	HA MNs	Shikonin	Targeted delivery	103
	Carboxymethyl cellulose MNs	Anti-TNF-α Ab	Targeted delivery	143
	HA, dextran, and PVP based layered MNs	TAC & diclofenac	Targeted delivery	107
**AD**	HA & PVP MNs	TAA	Enhanced penetration	144
	HA MNs	CRISPR-Cas9, dexamethasone	Targeted delivery	145
	Pγ-PGA & polycaprolactone MNs	γ-PGA	Targeted delivery	146
	γ-PGA MNs	EGCG	Targeted delivery	147
** Melanoma**	Bubble-generating MNs	Anti-CTLA-4	Enhanced penetration	92
	pH-responsive MNs	OVA	Controlled release	148
	H_2_O_2_-responsive MNs	CuO_2_ NPs	Controlled release	149
	Thermal SLN-packaged HA/PVP MNs	PTX & IR-780	Controlled release	150
	AuNCs & DOX-loaded HA-MNs	AuNCs & DOX	Controlled release	151
	HA MNs	aPD1 & 1-MT	Targeted delivery	152
	Vaccine MNs	Tumor cell lysates	Targeted delivery	153
	Cryogenic MNs fabricated by PBS supplemented with DMSO & sucrose	OVA	Targeted delivery	154
	Theranostics MNs	NIR950	Imaging, diagnosis, and therapeutics	155
	Integrated wearable bandage and MN electrochemical sensing platforms for tumor detecting	-	Imaging, diagnosis, and therapeutics	156
**Hemangioma**	SMN pretreatment	Propranolol & timolol	Enhanced penetration	157
	HA and PVP MNs	Propranolol	Enhanced penetration	158
**Alopecia**	VPA and CMC MNs	Valproic acid	Enhanced penetration	159
	Finasteride nanostructured lipid carriers-based HA MNs	Finasteride	Enhanced penetration	160
	Ceria nanozyme-integrated HA MNs	-	Targeted delivery	161
**Melasma**	PVP and methacrylic acid MNs	Tranexamic acid	Enhanced penetration	162
	PVP and PVA MNs	Tranexamic acid	Enhanced penetration	163
**Scar**	Hydroxypropyl-β-cyclodextrin-conjugated HA MNs	TAA	Enhanced penetration	164
	HA MNs	Bleomycin	Enhanced penetration	165
	HA MNs	Shikonin	Enhanced penetration	166

1-MT: 1-methyl-dl-tryptophan; AIEgen: aggregation-induced emission luminogen. CMC: carboxymethyl cellulose; DMSO: dimethyl sulfoxide; VPA: valproic acid.

**Table 3:** Currently active clinical trials with MNs for treating skin diseases.

Type of skin disease	Type of MNs	Loading drugs	Status	Phase	NCT identifier
Psoriasis	DMN	Calcipotriol-betamethasone dipropionate ointment	Unknown	Not applicable	NCT02955576
AGA	Solid MN	5% Minoxidil	Unknown	Phase 1	NCT02154503
Scars	Fractional radiofrequency microneedling device	-	Completed	Not applicable	NCT03380845
Scars	Solid MN	-	Unknown	Not applicable	NCT02025088
Scars	Microneedling radiofrequency device	-	Completed	Not applicable	NCT02207738
Acne vulgaris	Single radiofrequency microneedling device	-	Completed	Not applicable	NCT04213638
Facial pigmentation	DMN	-	Enrolling by invitation	Not applicable	NCT04583852
Facial pigmentation	Derma pen microneedling device	Trichloroacetic acid	Unknown	Not applicable	NCT03472235
Hyperhidrosis	Fractional MN radiofrequency	Botulinum toxin type A	Completed	Not Applicable	NCT03054480
Hyperhidrosis	Solid MN	Botulinum toxin type A	Completed	Phase 1	NCT03203174
Hyperhidrosis	Fractional MN radiofrequency	-	Completed	Not Applicable	NCT02823340
Birch pollen allergy	Solid MN	-	Completed	Phase 1	NCT01628484
Actinic keratosis	Stainless steel MN	Aminolevulinic acid	Completed	Not applicable	NCT02594644
Actinic keratosis	Solid MN	Aminolevulinic acid	Completed	Not applicable	NCT01812837
Actinic keratosis	Solid MN	Aminolevulinic acid	Completed	Phase 2	NCT02632110

Data obtained from http://www.clinicaltrials.gov.

**Table 4:** Currently active clinical trials with nanocarriers for treating skin diseases.

Type of skin disease	Type of nanocarriers	Loading drugs	Status	Phase	NCT identifier
Psoriasis vulgaris	Liposomes & ethosomes	Anthralin	Completed	Phase 4	NCT03348462
Psoriasis	MEs	MTX	Not yet recruiting	Phase 4	NCT04971239
Psoriasis	MEs	Cyclosporine A	Completed	Phase 3	NCT00438360
Lentigo maligna	Nanoemulsion	5-aminolevulinic acid	Completed	Phase 4	NCT02685592
AD	Liposomal gel	HL-009	Completed	Phase 2	NCT01568489
AD	MEs	DNK333	Completed	Phase 2	NCT01033097
Radiation induced dermatitis	Liposomes	APN201	Completed	Phase 2	NCT01513278
Actinic keratosis	Liposomes	T4N5	Unknown	Phase 3	NCT00002811
Actinic keratosis	Nanoemulsion	BF-200 ALA (Ameluz)	Completed	Phase 3	NCT02799069
Basal cell carcinoma	Liposomes	T4N5	Completed	Phase 2	NCT00089180

Data obtained from http://www.clinicaltrials.gov.

## Abbreviations

5-Fu: 5-Fluorouracil
AD: atopic dermatitis
AGA: androgenetic alopecia
aPD1: anti-PD1 antibody
AST: astaxanthin
ATRA: all-trans retinoic acid
AuNCs: gold nanocages
AuNPs: gold nanoparticles
AuNRs: gold nanorods
BMV: betamethasone valerate
cfDNA: cell-free DNA
CTLA-4: cytotoxic T-lymphocyte-associated antigen 4
DMN: dissolving microneedle
DOX: doxorubicin
DP: dermal papilla
DP-EVs: dermal papilla-derived extracellular vesicles
ECM: excessive extracellular matrix
EGCG: epigallocatechin gallate
EGF: epidermal growth factor
HA: hyaluronic acid
HFs: hair follicles
ICG: indocyanine green
ISF: interstitial fluid
MN: microneedle
MSNs: mesoporous silica nanoparticles
MTX: methotrexate
NIR: Near-infrared
NLRP3: the nod-like receptor pyrin domain associated protein 3
NPs: nanoparticles
OSA: oxidized sodium alginate
OVA: ovalbumin
PD-1: programmed cell death protein-1
PLGA: poly(lactic-co-glycolic) acid
PTT: photothermal therapy
PTX: paclitaxel
PVA: polyvinyl alcohol
PVP: polyvinylpyrrolidone
QDs: quantum dots
ROS: reactive oxygen species
SC: stratum corneum
SLN: solid lipid nanoparticle
SMN: Solid MNs
TAA: triamcinolone acetonide
TAC: tacrolimus
TD: transdermal enhanced peptide
TPL: triptolide
γ-PGA: poly-γ-glutamic acid
